# Fluorescent probe for the imaging of superoxide and peroxynitrite during drug-induced liver injury†

**DOI:** 10.1039/d0sc05937d

**Published:** 2021-01-04

**Authors:** Luling Wu, Jihong Liu, Xue Tian, Robin R. Groleau, Steven D. Bull, Ping Li, Bo Tang, Tony D. James

**Affiliations:** College of Chemistry, Chemical Engineering and Materials Science, Key Laboratory of Molecular and Nano Probes, Ministry of Education, Collaborative Innovation Center of Functionalized Probes for Chemical Imaging in Universities of Shandong, Institutes of Biomedical Sciences, Shandong Normal University Jinan 250014 People's Republic of China lip@sdnu.edu.cn tangb@sdnu.edu.cn; Department of Chemistry, University of Bath Bath BA2 7AY UK t.d.james@bath.ac.uk; School of Chemistry and Chemical Engineering, Henan Normal University Xinxiang 453007 P. R. China

## Abstract

Drug-induced liver injury (DILI) is an important cause of potentially fatal liver disease. Herein, we report the development of a molecular probe (**LW-OTf**) for the detection and imaging of two biomarkers involved in DILI. Initially, primary reactive oxygen species (ROS) superoxide (O_2_˙^−^) selectively activates a near-infrared fluorescence (NIRF) output by generating fluorophore **LW-OH**. The C=C linker of this hemicyanine fluorophore is subsequently oxidized by reactive nitrogen species (RNS) peroxynitrite (ONOO^−^), resulting in cleavage to release xanthene derivative **LW-XTD**, detected using two-photon excitation fluorescence (TPEF). An alternative fluorescence pathway can occur through cleavage of **LW-OTf** by ONOO^−^ to non-fluorescent **LW-XTD-OTf**, which can react further with the second analyte O_2_˙^−^ to produce the same **LW-XTD** fluorescent species. By combining NIRF and TPEF, **LW-OTf** is capable of differential and simultaneous detection of ROS and RNS in DILI using two optically orthogonal channels. Probe **LW-OTf** could be used to detect O_2_˙^−^ or O_2_˙^−^ and ONOO^−^ in lysosomes stimulated by 2-methoxyestradiol (2-ME) or 2-ME and SIN-1 respectively. In addition, we were able to monitor the chemoprotective effects of *tert*-butylhydroxyanisole (BHA) against acetaminophen (APAP) toxicity in living HL-7702 cells. More importantly, TPEF and NIRF imaging confirmed an increase in levels of both O_2_˙^−^ and ONOO^−^ in mouse livers during APAP-induced DILI (confirmed by hematoxylin and eosin (H&E) staining).

## Abstract 2

Drug-induced liver injury (DILI) is an important cause of potentially fatal liver disease.

## Graphical abstract

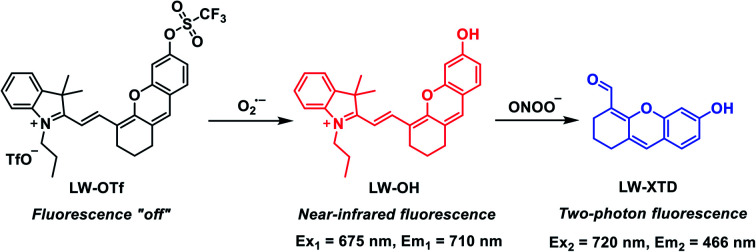

## Introduction

Drug-induced liver injury (DILI) is a leading cause of acute liver failure in the USA and Europe, and has as such raised serious concerns for public health.^1–3^ The potential to induce DILI is also one of the most common causes of compound attrition in drug development, often leading to drug withdrawals, restrictions, and project termination.^3^ Minimizing hepatotoxicity is therefore crucial, requiring effective techniques for preclinical screening of DILI.^4,5^ Unfortunately, probes capable of imaging DILI in living animals remain limited,^6–8^ making the development of methods for accurate diagnosis vital for improving the treatment of DILI.^9–11^

A common cause of DILI is overdose of acetaminophen (APAP), causing oxidative and nitrosative stress through elevated levels of reactive oxygen species (ROS) and reactive nitrogen species (RNS). Metabolism of APAP in the liver proceeds *via* transformation into a toxic metabolite, *N*-acetyl-*p*-benzoquinone imine (NAPQI).^12^ APAP hepatotoxicity is known to arise from the interference of NAPQI with complex I/II of the mitochondrial electron transport chain (ETC), resulting in the leakage of electrons from the ETC to oxygen which induces superoxide (O_2_˙^−^) formation.^13,14^ O_2_˙^−^ is then converted into hydrogen peroxide (H_2_O_2_) and oxygen (O_2_) by manganese superoxide dismutase, leading to additional oxidative stress. In addition, O_2_˙^−^ can react with endogenous nitric oxide (NO) to generate RNS peroxynitrite (ONOO^−^).^15^ Considering these ROS and RNS are products of different pathways, and exhibit different biological effects, their simultaneous detection could improve our understanding of the *in vivo* mode of action in DILI.^8,16–18^

Fluorescence imaging is commonly used as a non-invasive method to image and measure these types of analytes with high temporal and spatial resolution suitable for diagnostic applications in living organisms.^19^ Improvements to fluorescent methods can be made by using near-infrared fluorescence (NIRF, 650–900 nm), which benefits from minimized auto-fluorescence of endogenous biomolecules and reduced light scattering in tissues.^20–22^ While a number of NIRF-based probes have been used for the detection of O_2_˙^−^,^23–28^ none have yet been used to evaluate changes in O_2_˙^−^ in DILI. Further improvements to biological fluorescence imaging can be achieved with the use of two-photon excitation fluorescence (TPEF), which brings unique benefits such as increased spatial resolution and enhanced penetration depths.^29,30^ Nevertheless, such probes capable of two-photon excitation suitable for investigating the role of ONOO^−^ in DILI are still rare.^7^

**Scheme 1:**
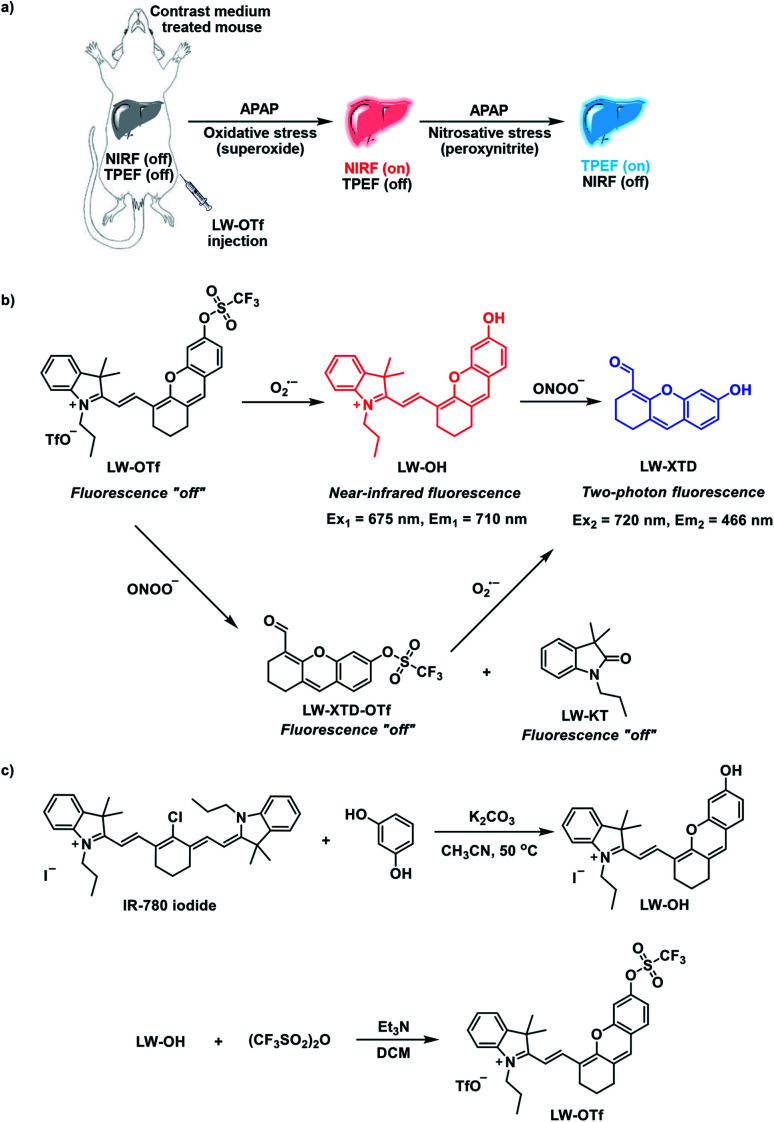
Design and synthesis of **LW-OTf**. (a) Duplex imaging and detection of DILI in mice using **LW-OTf**. (b) Sequential dual-response mechanisms of **LW-OTf** for O_2_˙^−^ and ONOO^−^. (c) Synthesis of target probe **LW-OTf**.

Our groups' research interests lie in developing new fluorescent probes for the detection and imaging of ROS and RNS, with a recent focus on dual-response probes.^30–34^ Carrying on this work, fluorescent probe **LW-OTf** was designed and synthesized with the intention imaging of DILI. O_2_˙^−^, a primary ROS, and ONOO^−^, a prominent RNS, were chosen as pertinent DILI-related biomarkers for this study. To the best of our knowledge, **LW-OTf** represents the first reaction-based small-molecule fluorescent probe having NIRF and TPEF capabilities with two independent optical channels: NIRF for O_2_˙^−^ and TPEF for ONOO^−^ (Scheme 1a and b).

The synthesis of **LW-OTf** was carried out over two steps (Scheme 1c and S3†). Hemicyanine-based fluorophore **LW-OH** was first prepared by *retro*-Knoevenagel reaction^35^ followed by addition of a O_2_˙^−^-reactive trifluoromethylsulfonyl unit (triflyl, Tf) to afford **LW-OTf **^36,37^ (for further discussion of the TfO^−^ counteranion see the ESI†). In the presence of O_2_˙^−^ triflyl deprotection occurs, which leads to an increased NIRF signal by generation of fluorophore **LW-OH**. Subsequent reaction with ONOO^−^ results in oxidative cleavage of the alkene linker of **LW-OH** to generate xanthene derivative **LW-XTD**,^38–40^ capable of two-photon fluorescence (see ESI† for further discussion of hemicyanine-xanthene fluorescent turn-on mechanism and selectivity). An alternative fluorescence activation pathway can also occur, in which **LW-OTf** is first cleaved by ONOO^−^ to produce non-fluorescent **LW-XTD-OTf**, which can subsequently react with superoxide to produce the same final xanthene derivative **LW-XTD**. Unfortunately, fluorescence experiments could not detect this second pathway, as generation of ONOO^−^ requires an aqueous medium, which led to rapid decomposition of KO_2_-derived O_2_˙^−^ in an assay setting, and so sequential addition of peroxyntrite then superoxide could not be carried out.^41^ This dual-response molecular design allows **LW-OTf** to produce either NIRF or TPEF signals in response to O_2_˙^−^ and ONOO^−^, respectively (Scheme 1b).

## Results and discussion

**Fig. 1:**
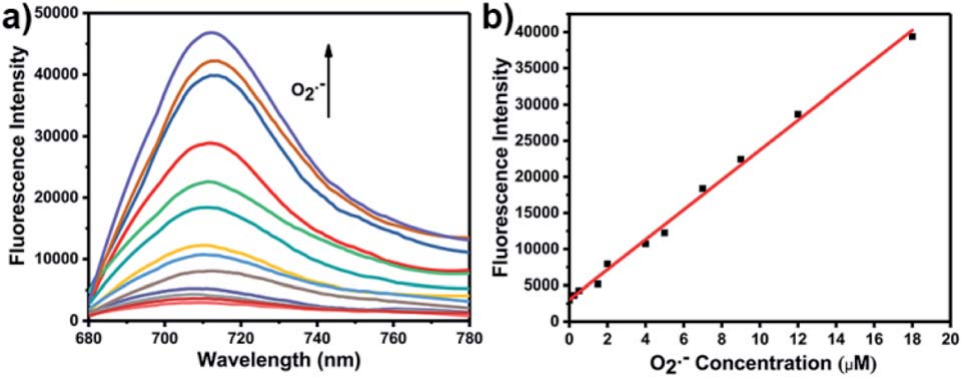
(a) One-photon fluorescence spectra of **LW-OTf** (2.4 μM) after addition of KO_2_ (0–25 μM). (b) Linear relationship between fluorescence intensity of **LW-OTf** (2.4 μM) and concentration of O_2_˙^−^ (0–18 μM). *λ*_ex/em_ = 675/710 nm. Note: O_2_˙^−^ was prepared by dissolving KO_2_ in DMSO, and was then added to **LW-OTf** (in DMSO). The mixture was diluted with PBS buffer (10 mM, pH 7.4) before each measurement. See ESI for detailed procedures.†

**Fig. 2:**
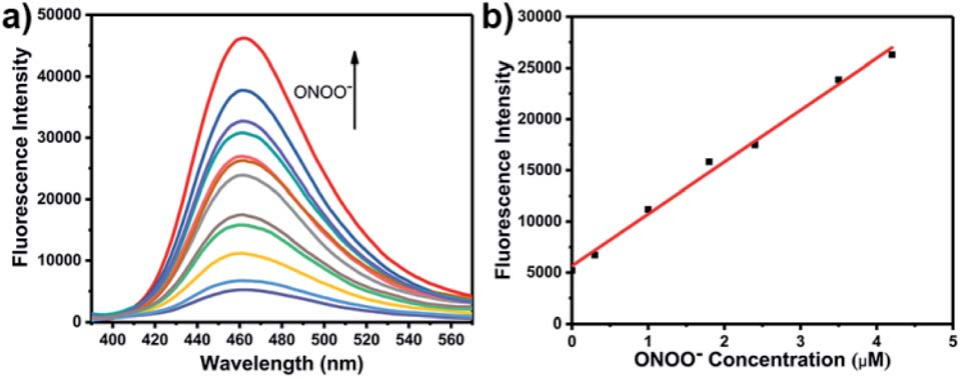
(a) One-photon fluorescence spectra of **LW-OTf** (2.4 μM) pre-incubated with O_2_˙^−^ (5 μM), followed by addition of ONOO^−^ (0–36 μM). *λ*_ex/em_ = 360/461 nm. (b) Linear relationship between fluorescence intensity of **LW-OTf** (2.4 μM, pre-incubated with O_2_˙^−^ (5 μM)) and concentration of ONOO^−^ (0–4.2 μM). *λ*_ex/em_ = 360/461 nm. Note: O_2_˙^−^ was prepared by dissolving KO_2_ in DMSO, and was then added to **LW-OTf** (in DMSO), followed by addition of ONOO^−^ (in water). The mixture was diluted with PBS buffer (10 mM, pH 7.4) before each measurement. See ESI for detailed procedures.†

With **LW-OTf** in hand, we first evaluated its optical properties. As shown in Fig. S1,†**LW-OTf** (10 μM, pH 7.4) exhibited absorption maxima at 546 and 576 nm, whilst for **LW-OH** (formed *in situ* by addition of O_2_˙^−^, 20 μM) those absorptions decreased, with a new maximum at 687 nm. Subsequent addition of ONOO^−^ (17.5 μM) to the solution resulted in the emergence of a peak at 353 nm. These observations are in good agreement with the proposed mechanism for the sequential reaction of **LW-OTf** with O_2_˙^−^ followed by ONOO^−^ (Scheme 1). The fluorescence behavior (Fig. 1 and 2) of this sensing system was then evaluated. Initially, negligible fluorescence was observed, with incremental addition of O_2_˙^−^ (0–25 μM) causing a continuous increase in emission intensity at 710 nm using excitation at 675 nm (Fig. 1a). Removal of the triflyl unit in the presence of O_2_˙^−^ released fluorophore **LW-OH**, causing a 15.6-fold enhancement in fluorescence emission intensity. An excellent linear relationship between the emission intensity at 710 nm and the concentration of O_2_˙^−^ over the 0–18 μM range was observed (linear equation: *y*_1_ = 3030 + 2068 × [O_2_˙^−^] (μM), *R*^2^ = 0.995, *y*_1_ is the intensity at 710 nm, Fig. 1b), and the detection limit was calculated to be 46.5 nM. The fluorescence behavior of **LW-OTf** in the presence of both O_2_˙^−^ and ONOO^−^ was then evaluated. Excitation at 360 nm was selected for one-photon fluorescence experiments, matching the observed maximum absorption for **LW-XTD** at 353 nm, as well as previous reports of this system.^38^**LW-OTf** initially exhibited a weak emission signal at 461 nm in the presence of O_2_˙^−^, however upon subsequent addition of ONOO^−^ the D–π–A-based oxidation product **LW-XTD** exhibited a strong fluorescence emission at 461 nm, upon excitation at 360 nm (Fig. 2a). As the concentration of ONOO^−^ was increased from 0 to 4.2 μM the fluorescence intensity at 461 nm gradually increased as well (linear equation: *y*_2_ = 5657 + 5074 × [ONOO^−^] (μM), *R*^2^ = 0.994, *y*_2_ is the intensity at 461 nm), with a detection limit for ONOO^−^ of 38.2 nM (Fig. 2b). The use of two-photon microscopy, was first described by Webb *et al.* in 1990, and has since been adopted for bioimaging applications.^42^ Given that the excitation of a fluorophore using two-photon fluorescence is twice that of one-photon fluorescence, an excitation wavelength of 720 nm was chosen for two-photon measurements. **LW-OTf** also exhibited two-photon fluorescence for the detection of ONOO^−^ in the presence of O_2_˙^−^*in vitro* using an excitation of 720 nm (Fig. S2†).

**Fig. 3:**
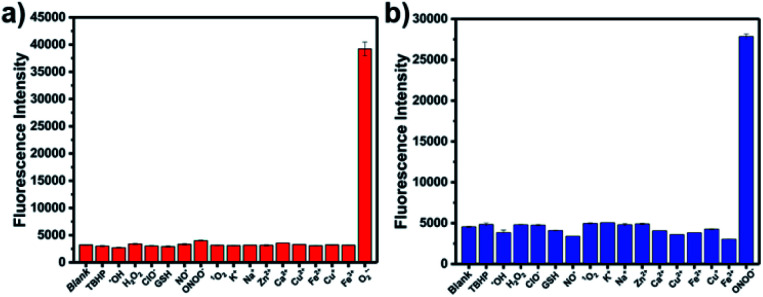
(a) Red fluorescence response of **LW-OTf** (2.4 μM) to various reactive oxygen species/reactive nitrogen species or metals ions (100 μM TBHP, 100 μM ˙OH, 10 mM H_2_O_2_, 100 μM NaClO, 10 μM GSH, 50 μM NO˙, 25 μM ONOO^−^, 100 μM ^1^O_2_, 10 mM K^+^, 10 mM Na^+^, 1 mM Zn^2+^, 1 mM Ca^2+^, 100 μM Cu^2+^, 100 μM Fe^2+^, 100 μM Cu^+^, 100 μM Fe^3+^, and 18 μM O_2_˙^−^). *λ*_ex/em_ = 675/710 nm. (b) One-photon blue fluorescence responses of probe **LW-OTf** (2.4 μM) to addition of O_2_˙^−^ (5 μM) followed by addition of various reactive oxygen species/reactive nitrogen species or metal ions (100 μM TBHP, 100 μM ˙OH, 10 mM H_2_O_2_, 100 μM NaClO, 10 μM GSH, 50 μM NO˙, 100 μM ^1^O_2_, 10 mM K^+^, 10 mM Na^+^, 1 mM Zn^2+^, 1 mM Ca^2+^, 100 μM Cu^2+^, 100 μM Fe^2+^, 100 μM Cu^+^, 100 μM Fe^3+^, and 4.2 μM ONOO^−^). *λ*_ex/em_ = 360/461 nm.

The optical selectivity of **LW-OTf** towards the selected ROS and RNS was then evaluated *in vitro*, confirming that its fluorescence response was most sensitive to the presence of O_2_˙^−^ (Fig. 3a). As previously noted, C=C cleavage by ONOO^−^ could also occur, however as this does not lead to a fluorescent signal, no selectivity issues arose. Selectivity towards ONOO^−^ was then determined using a stepwise approach, first incubating **LW-OTf** with superoxide, then adding either ONOO^−^ or a range of other ROS. Fragmentation of **LW-OH** to **LW-XTD** resulting from RNS-mediated oxidative cleavage of the C=C linker was monitored by measuring the increase in emission at 461 nm after excitation at 360 nm. These experiments demonstrated that intermediate **LW-OH** was specifically responsive to ONOO^−^ over H_2_O_2_, NO˙, ˙OH, ^1^O_2_, and ClO^−^ (Fig. 3b). pH titrations indicated that the fluorescence intensity of **LW-OTf** was greatest at pH 7–8, matching the physiological pH at which this probe would operate *in vivo* (Fig. S3 and S5†). Fluorescence intensities at 710 nm decreased significantly at lower pH, likely due to phenolic protonation of **LW-OH**, resulting in reduced intramolecular charge transfer (ICT) (Fig. S3†).^43–45^ Decomposition of ONOO^−^ at acidic pH is likely responsible for the decreased emission intensities at 461 nm (Fig. S5†).^46^

In order to better understand and ultimately confirm the suggested mode of action of probe **LW-OTf**, we assessed the time course of the reaction of **LW-OTf** with both O_2_˙^−^ and ONOO^−^ (Fig. S4 and S6†). Whilst the reaction of **LW-OTf** with O_2_˙^−^ was finished within 10 min (Fig. S4†), the subsequent addition of ONOO^−^ resulted in an instantaneous and significant fluorescence increase (Fig. S6†). Both reaction profiles are consistent with the known reactivity of both analytes, and offer promising prospects for future applications, since rapid detection is particularly important for real-time detection of O_2_˙^−^ and ONOO^−^ in living systems. In addition, high-resolution LC-MS experiments were performed to confirm the proposed fluorescence mechanisms (Fig. S7–S12†). Addition of KO_2_ (3 equiv. in DMSO) to a solution of **LW-OTf** (in MeOH) resulted in the representative cation of **LW-OH** ([M]^+^, *m*/*z* = 412.2283), indicating the triflyl-deprotection of **LW-OTf** by superoxide (Fig. S8†). The mass spectra following the addition of ONOO^−^ (1 equiv. in water) were found to be consistent with the mechanism proposed above, with detection of the mass ion for **LW-XTD** ([M + H]^+^, *m*/*z* = 229.0860) as well as the indoline by-product confirming oxidative cleavage of **LW-OH** by ONOO^−^ (Fig. S9 and S10†).^38,47^ HRMS was also used to prove the alternate activation pathway, with direct addition of ONOO^−^ (5 equiv. in water) to **LW-OTf** (in MeOH) generating a mass ion for **LW-XTD-OTf** ([M + H]^+^, *m*/*z* = 361.0357, Fig. S12†).^39,40^

These promising *in vitro* results prompted us to explore the duplex imaging of **LW-OTf** in living cells and in mice. Using MTT assays, it was confirmed that **LW-OTf** was non-toxic to HL-7702 cells (Fig. S13†). Pre-treatment of the cells with a superoxide scavenger Tiron (10 μM, 30 min),^48,49^ followed by incubation with **LW-OTf** (2.4 μM) for a further 15 min resulted in only weak fluorescence in the red channel (Fig. S14a†). Conversely, pre-treatment with Tiron followed by stimulation by varying amounts of 2-methoxyestradiol^50–52^ (2-ME, 0, 0.5, 2.0, 3.0 μg mL^−1^), an O_2_˙^−^ promoter, resulted in significant fluorescence intensity enhancement in the red channel (Fig. S14a†). Only weak fluorescence and no significant change was observed in the blue channel, for which an excitation wavelength of 405 nm was used (closest available to 360 nm). These results clearly confirm the ability of **LW-OTf** to selectively detect superoxide in cells using NIRF.

Interestingly, **LW-OTf** detected O_2_˙^−^ primarily in lysosomes after stimulation with 2-ME (red channel, Fig. S16a, d, g and j†), probably facilitated by endocytosis.^45^ Co-staining with commercial organelle markers was performed using Lyso-Tracker Green (Fig. S16b†), ER-Tracker Red (Fig. S16e†), Golgi-Tracker Red (Fig. S16h†) and Mito-Tracker Green (Fig. S16k†). The Pearson correlation coefficients with the markers for the lysosomes, endoplasmic reticulum, Golgi, and mitochondria were 0.87, 0.51, 0.39 and 0.35, respectively.

As shown in Fig. S14b,† cells were first pre-incubated with Tiron (10 μM), then exposed to 2-ME (3.0 μg mL^−1^), followed by staining with **LW-OTf** (2.4 μM), and finally incubated with SIN-1 (ONOO^−^ donor).^53^ A concentration-dependent change in fluorescence emission intensity in the blue and red channel was observed. The addition of 3.0 mM SIN-1 led to a 4.25-fold enhancement of the average blue fluorescence intensity and 2.54-fold decrease in the average red fluorescence intensity (Fig. S14d†). Similar to the results shown in Fig. S16, **LW-OTf** demonstrated the ability to visualize ONOO^−^ in lysosomes with a Pearson correlation coefficient of 0.90 (Fig. S17†).

**Fig. 4:**
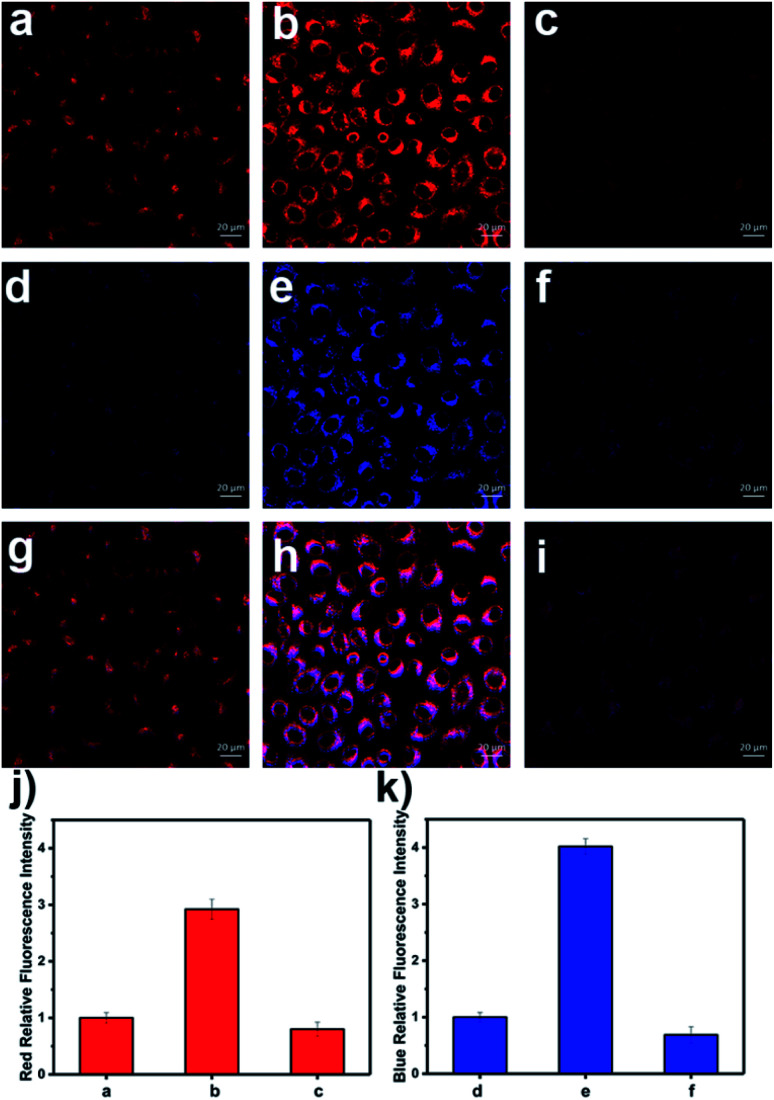
NIRF and TPEF images of APAP-induced injury of HL-7702 cells. (a, d and g) Cells were stained with probe **LW-OTf** (2.4 μM) for 15 min. (b, e and h) Cells were incubated with APAP (20 mM) for 1 h, and then stained with **LW-OTf** (2.4 μM) for 15 min. (c, f and i) Cells were pretreated with BHA (500 μM) for 1 h, then incubated with APAP (20 mM) for 1 h, followed by staining with **LW-OTf** (2.4 μM) for 15 min. (a–c) Red fluorescence channel for O_2_˙^−^: *λ*_ex_ = 633 nm, *λ*_em_ = 635–747 nm; (d–f) Blue fluorescence channel for ONOO^−^: *λ*_ex_ = 720 nm, *λ*_em_ = 420–550 nm. (g–i) Merged fluorescence channels. (j) Red relative fluorescence intensity output of (a–c). (k) Blue relative fluorescence intensity output of (d–f). The fluorescence intensity of the control group is defined as 1.0. The data are expressed as the mean ± SD. Similar results were obtained in quintuplicate.

Since overdose of APAP leads to the overproduction of ROS and RNS,^54^ APAP-induced DILI was chosen as a representative model for liver toxicity in which to evaluate the effectiveness of **LW-OTf**. Although this model is often used for single detection of ONOO^−^,^7,55–57^ simultaneous detection of RNS and ROS in DILI is still rare.^8^ Treatment of HL-7702 cells with APAP and **LW-OTf** produced a marked increase in fluorescence in both the red and blue channels (Fig. 4b and e), indicating upregulation of intracellular O_2_˙^−^ and ONOO^−^ after administration of APAP, and demonstrating the ability of our probe to detect concentration changes of O_2_˙^−^ by NIRF and ONOO^−^ by TPEF in DILI. This was further confirmed using *tert*-butylhydroxyanisole (BHA), a ROS and RNS scavenger^58^ which has been used to eliminate ROS and relieve APAP-induced liver injury.^59,60^ Upon addition of BHA, the fluorescence intensity for both the red and blue channels decreased (Fig. 4c and f). Similarly, **LW-OTf** exhibited the expected one-photon fluorescence changes in the blue and red channel from APAP-induced hepatotoxicity and remediation using BHA (Fig. S15†).

**Fig. 5:**
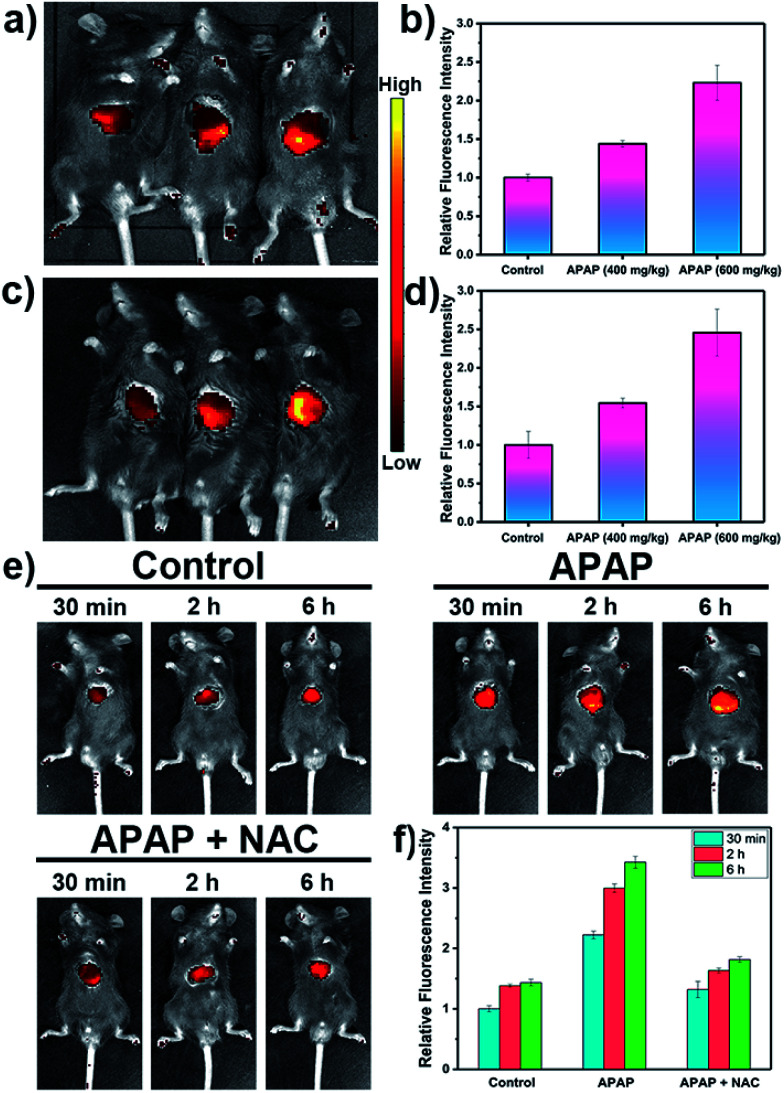
NIRF imaging of APAP-induced injury *in vivo*. (a) After surgical treatment, the liver of each mouse was exposed for *in vivo* imaging. (c) After intraperitoneal injection of **LW-OTf** (200 μL, 48 μM), the mice were depilated to evaluate deep tissue penetration of the probe **LW-OTf**. (a and c) Fluorescence imaging of C57 mice in the control group (APAP 200 mg kg^−1^, left), and APAP-induced injury model groups(APAP 400 mg kg^−1^, middle; APAP 600 mg kg^−1^, right), followed by intraperitoneally injection of **LW-OTf** (200 μL, 48 μM). (b and d) Red relative fluorescence intensity output of all three groups (a and c, respectively). The fluorescence intensity of the control group is defined as 1.0. (e) Fluorescence imaging of control, APAP (600 mg kg^−1^), and APAP (600 mg kg^−1^) with NAC (400 mg kg^−1^). Imaging carried out 30 min, 2 h, 6 h after APAP injection. (f) Relative fluorescence intensity in (e) and the fluorescence intensity of the control group (30 min) is defined as 1.0. Mice imaging was carried out in the red channel: *λ*_ex/em_ = 660/710 nm. The data are expressed as the mean ± SD. Five mice in each group.

Inspired by these cell imaging experiments, **LW-OTf** was used for *in vivo* imaging of O_2_˙^−^ and ONOO^−^ in DILI. Towards that aim, C57 mice were treated with APAP either at analgesic low dosage (200 mg kg^−1^, control group), or high dosages (400 mg kg^−1^ and 600 mg kg^−1^). After 6 h,^61,62^ all three groups were given intraperitoneal injections of **LW-OTf** (200 μL, 48 μM), and imaging was performed after a further 15 min. The NIRF imaging ability for O_2_˙^−^ in exposed livers was investigated first. As shown in Fig. 5a (left mouse) and Fig. 5b, only weak fluorescence at 710 nm was observed in the control group mice, implying only low concentrations of O_2_˙^−^ for low doses of APAP. In contrast, after the administration of high doses of APAP, the mouse livers displayed significant fluorescence enhancements in a concentration-dependent manner, indicating DILI-induced overproduction of O_2_˙^−^ after APAP treatment. Deep tissue penetration imaging of O_2_˙^−^ in depilated mice using probe **LW-OTf** indicated a 1.54-fold (APAP 400 mg kg^−1^) and 2.46-fold (APAP 600 mg kg^−1^) increase in the emission at 710 nm (Fig. 5c and d). Furthermore, as displayed in Fig. 5e and f, the highest dosage group (APAP 600 mg kg^−1^) showed significant fluorescence enhancement over time, indicating continued DILI-induced ROS overproduction. To confirm this, *N*-acetylcysteine (NAC), a hepatoprotective agent, was injected into the DILI mice (APAP 600 mg kg^−1^), resulting in a significant attenuation in the fluorescent signal back to levels comparable to the control group mice.

**Fig. 6:**
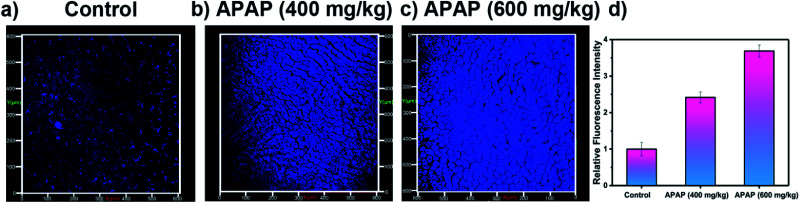
Two-photon fluorescence images of APAP-induced injury *in vivo*, 3D images of the livers of mice incubated with **LW-OTf** (48 μM) for 15 min. (a) Control group. (b and c) Mice were intraperitoneally injected with APAP (400 mg kg^−1^, 600 mg kg^−1^, respectively), followed by **LW-OTf** (200 μL, 48 μM). (d) Blue relative fluorescence intensity output of all the three groups. The fluorescence intensity of the control group is defined as 1.0. Mice imaging was carried out in the blue channel: *λ*_ex_ = 720 nm, *λ*_em_ = 397–571 nm. The data are expressed as the mean ± SD. Five mice in each group.

Our attention then turned to two-photon *in vivo* bio-imaging of ONOO^−^ using **LW-OTf**, using the same mouse models as discussed above. Following surgical treatment, the liver of each mouse was assessed using two-photon fluorescence imaging with excitation at 720 nm. The livers of mice under nitrosative stress (Fig. 6c) exhibited distinct fluorescence enhancements (approximately 3.68-fold) when compared to the control mice (Fig. 6a). These results validate the ability of **LW-OTf** to image both superoxide and peroxynitrite *in vivo*, using NIRF for the former, and TPEF for the latter.

**Fig. 7:**
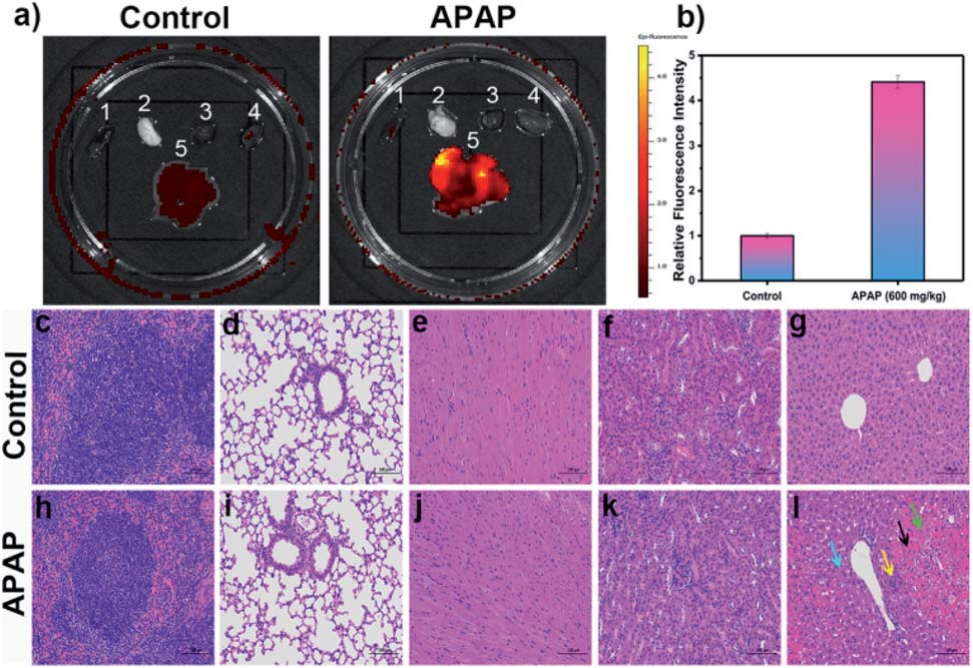
(a) Fluorescence imaging in the major organs isolated from control group and model group with APAP (600 mg kg^−1^)-induced liver injury. 1: spleen, 2: lung, 3: heart, 4: kidney, 5: liver. (b) Relative fluorescence intensity output of livers isolated from two groups. The fluorescence intensity of control group is defined as 1.0. Mice organ imaging was operated only in the red channel: *λ*_ex/em_ = 660/710 nm. The hematoxylin and eosin (H&E) staining of main organs in mice (c–l). (c–g) Spleen, lung, heart, kidney, liver tissue section of control group. (h–l) Spleen, lung, heart, kidney, liver tissue section of model group with APAP (600 mg kg^−1^)-induced liver injury. (g) The structure of the liver lobules was clear, the hepatocytes were arranged neatly. No obvious degeneration and necrosis of hepatocytes were observed. There was no obvious congestion of hepatic sinusoid. No obvious inflammatory cell infiltration was seen. (l) Compared to the control group, there were obvious liver damages in the model group: significant congestion and hemorrhage in the sinusoids of the acinar III zone (black arrow); a large amount of hepatocyte steatosis with dense vacuoles in the cytoplasm (blue arrow); some hepatocytes with dissolved and disappeared nucleus were necrotic (green arrow); slight inflammatory infiltration and individual inflammatory foci in the liver lobules (yellow arrow). Scale bar = 100 μm. The data are expressed as the mean ± SD. Five mice in each group.

Following on from these *in vivo* results, we wished to explore the distribution of DILI-induced ROS/RNS in mice. Again, DILI model group mice were injected intraperitoneally with a dose of APAP (600 mg kg^−1^), whilst a control group was given only physiological saline. After 6 h both groups were injected with **LW-OTf** (200 μL, 48 μM) and left for 15 min before being killed and dissected to isolate their major organs for *ex vivo* NIRF imaging (Fig. 7a and b). As shown in Fig. 7a, when compared to the other organs (spleen, lung, heart, and kidney), a significant fluorescence signal was observed in the liver of both DILI and control group mice. In addition, **LW-OTf** exhibited a stronger fluorescence signal in the livers of DILI mice than in those of healthy mice (Fig. 7b). Furthermore, hematoxylin and eosin (H&E) staining of the liver tissues and other major organ tissues (spleen, lung, heart, and kidney) was carried out to identify the histological changes during the APAP treatment. All tissue types from both the control and model groups were examined for tissue architecture, degeneration, necrosis, hemorrhage, and inflammatory cell infiltration, looking for splenic, pulmonary, cardiac, renal, and hepatic damage. No obvious differences or damage were observed upon comparison of the control and model tissue samples of the spleen, lung, heart and kidney, indicating clearly that no APAP-induced damage had occurred (Fig. 7c–f and h–k).

As displayed in Fig. 7g, the control group liver appeared healthy, with the structure of the liver lobules clear, neatly arranged hepatocytes, and no obvious degeneration, necrosis, inflammatory cell infiltration or congestion of hepatic sinusoids. The hepatocytes from the model group (Fig. 7l), on the other hand, exhibited clear signs of liver damage. Significant congestions and hemorrhage was visible in the sinusoids of the acinar III zone (black arrow), and a large amount of hepatocyte steatosis with dense vacuoles in the cytoplasm (blue arrow) were observed. Some hepatocytes with dissolved and disappeared nuclei were necrotic (green arrow). Slight inflammatory infiltration and individual inflammatory foci in the liver lobules could be seen (yellow arrow). Thus, these H&E staining results are in good agreement with the results of *in vitro* and *in vivo* fluorescence imaging using O_2_˙^−^ and ONOO^−^ as biomarkers.

## Conclusions

In conclusion, we have established **LW-OTf** as the first molecular fluorescent probe for the *in situ* imaging of RNS and ROS associated with drug-induced liver injury in living cells and mice. **LW-OTf** was able to detect O_2_˙^−^ and ONOO^−^*via* near-infrared fluorescence and two-photon fluorescence, respectively. We believe that the molecular design of **LW-OTf** can be generalized for dual imaging of other biomarkers (*e.g.* alkaline phosphatase)^63^ and ONOO^−^ in DILI by simply changing the protecting group on the NIRF signaling moiety. In principle, the detection of RNS and ROS in real time with fluorescence imaging agents could significantly help guide the understanding of ROS- and RNS-related diseases and potentially contribute to the development of new approaches for the treatment of DILI.

## Conflicts of interest

There are no conflicts to declare.

## Supplementary Material

SC-012-D0SC05937D-s001
