# Supplementary material for: Fluorescent probe for the imaging of superoxide and peroxynitrite during drug-induced liver injury
Source: Chem Sci. 2021 Jan 4;12(11):3921–8. doi: 10.1039/d0sc05937d (PMC8179478; doi:10.1039/d0sc05937d)
Supplement: SC-012-D0SC05937D-s001 [file SC-012-D0SC05937D-s001.pdf]

Electronic Supplementary Material (ESI) for Chemical Science.
This journal is © The Royal Society of Chemistry 2021

Supporting Information

Fluorescent Probe for the Imaging of Superoxide and Peroxynitrite during Drug-induced Liver Injury

Luling Wu,[‡a,b] Jihong Liu,[‡a,b] Xue Tian,[b] Robin R. Groleau,[b] Steven D. Bull,[b] Ping Li,*[a] Bo Tang,*[a] and Tony D. James*[a,b,c]

[a]College of Chemistry, Chemical Engineering and Materials Science, Key Laboratory of Molecular and Nano Probes, Ministry of Education, Collaborative Innovation Center of Functionalized Probes for Chemical Imaging in Universities of Shandong, Institutes of Biomedical Sciences, Shandong Normal University, Jinan 250014, People's Republic of China.

[b]Department of Chemistry, University of Bath, Bath, BA2 7AY, UK.

[c]School of Chemistry and Chemical Engineering, Henan Normal University, Xinxiang 453007, P. R. China.

[‡]Equal contribution

Email: lip@shdu.edu.cn; tangb@shdu.edu.cn; t.d.james@bath.ac.uk

Table of contents

1. Materials and instruments S2
2. Preparation of ROS/RNS S2
3. UV-Vis and fluorescence analysis S3
4. Mass spectroscopic analysis S8
5. Protocols for cell culture S23
6. Fluorescence imaging in live cells and MTT assay S23
7. Near-infrared fluorescence imaging of APAP-induced injury in mice S30
8. Two-photon fluorescence imaging of APAP-induced injury in mice S30
9. Hematoxylin and eosin (H&E) staining of main organs in mice S30
10. Synthesis of probe LW-OTf S32
11. NMR spectra S34
12. Characterisation of LW-OTf triflate salt S36
13. Fluorescence "turn on" mechanisms and selectivity of LW-OTf and other hemicyanine-xanthene dyes S40
14. Statistical analysis S40
15. References S41

1. Materials and instruments

2-Methoxyestradiol (2-ME) was purchased from Aladdin Industrial Corporation. 3-Morpholinosydnonimine hydrochloride (SIN-1) was purchased from Sigma-Aldrich Co. LLC. 4-Acetamidophenol (APAP) and butylated hydroxyanisole (BHA) were purchased from Shanghai Aladdin Biochemical Technology Co., Ltd. LC- MS analysis were performed using an Agilent QTOF 6545 with Jetstream ESI spray source coupled to an Agilent 1260 Infinity II Quat pump HPLC with 1260 autosampler, column oven compartment and variable wavelength detector (VWD). Fluorescence spectra were obtained with a FLS-1000-SS-STM spectrometer (Edinburgh Instruments Ltd., England). Absorption spectra were recorded on a BMG Labtech CLARIOstar®. MTT assay was performed using a Triturus microplate reader. Confocal imaging was performed on Leica SP8 high-resolution fluorescence microscope. The two-photon fluorescence images were taken using a LSM 880 confocal laser scanning microscope (Zeiss Co., Ltd. Germany). Two-photon fluorescence response in Figure S2 was performed with Zeiss 880 NLO microscope and the supporting software ZEN 2 lite using "Lambda Mode" in imaging setup. Fluorescence imaging in mice was performed on a Caliper IVIS Lumina Series III. Hematoxylin-eosin (H&E) staining images were obtained with optical microscope (NIKON Eclipse ci). Nuclear Magnetic Resonance (NMR) spectroscopy experiments were performed in deuterated solvent at 298 K on either a Bruker Avance 500 MHz spectrometer or an Agilent ProPulse 500 MHz spectrometer.

2. Preparation of ROS/RNS

$O_2^{\bullet-}$

$O_2^{\bullet-}$ was produced from KO_2 in dry DMSO by an ultrasonic method. The concentration of $O_2^{\bullet-}$ was determined from the absorption at 250 nm ($\varepsilon = 2682\ M^{-1}\ cm^{-1}$).

$ONOO^-$

0.6 M $NaNO_2$, 0.6 M HC1 and 0.7 M H_2O_2 were added simultaneously to a 3 M NaOH solution at 0 ºC. The concentration of peroxynitrite was determined using extinction coefficient of 1670 $M^{-1}\ cm^{-1}$ at 302 nm in 0.1 M NaOH (aq.).

H_2O_2

H_2O_2 solutions were accessed by dilution of 30% hydrogen peroxide aqueous solution, the concentration was determined from the absorption at 240 nm ($\varepsilon = 43.6\ M^{-1}\ cm^{-1}$).

TBHP

TBHP solutions were accessed by dilution of 70 % *tert*-butyl hydroperoxide aqueous solution.

$^{\bullet}OH$

$^{\bullet}OH$ (hydroxyl radical) was generated by the Fenton reaction of $FeCl_2$ (1.0 mM) and H_2O_2 (200 μM) in deionised water.

NO

NO (Nitric oxide) was obtained from a stock solution prepared by sodium nitroprusside.

$^1O_2$

$^1O_2$ (Singlet oxygen) was prepared by the NaClO-H_2O_2 system.

3. UV-Vis and fluorescence analysis

3.1 Experimental procedure for fluorescence measurement of $O_2^{\bullet-}$ (Figure 1).

Various concentrations of $O_2^{\bullet-}$ (0–25 μM) were added to probe **LW-OTf** in DMSO. After 1 min the mixture was diluted to 1 mL with PBS buffer (10 mM, pH 7.4) and incubated for 10 min before measurements. The final concentration of **LW-OTf** in PBS buffer solution (with 1% DMSO) was 2.4 μM. $\lambda_{ex/em}$ = 675/710 nm.

3.2 Experimental procedure for fluorescence measurement of $ONOO^-$ (Figure 2).

$O_2^{\bullet-}$ (5 μM) was added to probe **LW-OTf** in DMSO. After 1 min, various concentrations of $ONOO^-$ (0–36 μM) were added to the solution, and after an additional 1 min the mixture was diluted to 1 mL with PBS buffer (10 mM, pH 7.4) and incubated for 7 min. The final concentration of **LW-OTf** in PBS buffer solution (with 1% DMSO) was 2.4 μM. $\lambda_{ex/em}$ = 360/461 nm

3.3 Experimental procedures for absorption spectra (Figure S1).

Probe: Probe **LW-OTf** (10 μM) was diluted to 1 mL with PBS buffer (10 mM, pH 7.4, 1% DMSO).

Probe + 20 μM $O_2^{\bullet-}$: $O_2^{\bullet-}$ (20 μM) was added to **LW-OTf** in DMSO, and after 1 min the mixture was diluted to 1 mL with PBS buffer (10 mM, pH 7.4) and incubated for 10 min. The final concentration of **LW-OTf** in PBS buffer solution (with 1% DMSO) was 10 μM.

Probe + 20 μM $O_2^{\bullet-}$ + 17.5 μM $ONOO^-$: $O_2^{\bullet-}$ (20 μM) was added to **LW-OTf** in DMSO, and after 1 min $ONOO^-$ (17.5 μM) was added. After an additional 1 min the mixture was diluted to 1 mL with PBS buffer (10 mM, pH 7.4) and incubated for 7 min. The final concentration of **LW-OTf** in PBS buffer solution (with 1% DMSO) was 10 μM.

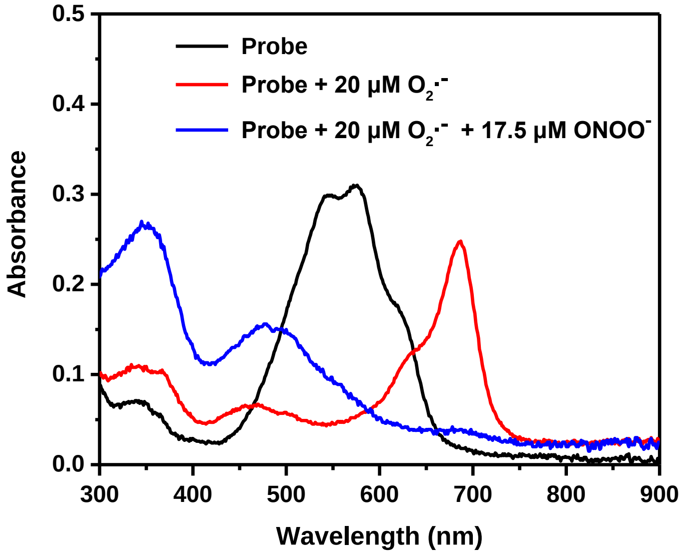

Figure S1. Absorption spectra of probe **LW-OTf** (10 μM) with and without $O_2^{\bullet-}$ (20 μM), and **LW-OTf** (10 μM) with the addition of $O_2^{\bullet-}$ (20 μM) then addition of $ONOO^-$ (17.5 μM).

3.4 Experimental procedures for two-photon experiments (Figure S2).

Probe + 5 µM $O_2^{\bullet-}$: $O_2^{\bullet-}$ (5 µM) was added to probe **LW-OTf** in DMSO, and after 1 min the mixture was diluted to 1 mL with PBS buffer (10 mM, pH 7.4) and incubated for 10 min. The final concentration of **LW-OTf** in PBS buffer solution (with 1% DMSO) was 2.4 µM.

Probe + 5 µM $O_2^{\bullet-}$ + 4.2 µM $ONOO^-$: $O_2^{\bullet-}$ (5 µM) was added to probe **LW-OTf** in DMSO, and after 1 min $ONOO^-$ (4.2 µM) was added. After an additional 1 min the mixture was diluted to 1 mL with PBS buffer (10 mM, pH 7.4) and incubated for 7 min. The final concentration of **LW-OTf** in PBS buffer solution (with 1% DMSO) was 2.4 µM.

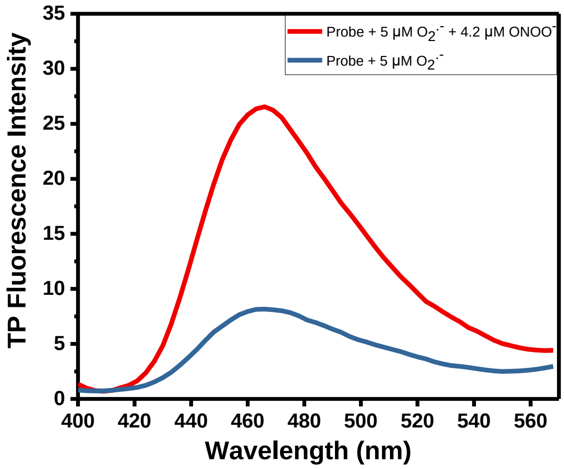

Figure S2. Two-photon fluorescence response of probe **LW-OTf** (2.4 µM) with $O_2^{\bullet-}$ (5 µM), and **LW-OTf** (2.4 µM) with the addition of $O_2^{\bullet-}$ (5 µM) then addition of $ONOO^-$ (4.2 µM). λ_{ex} = 720 nm, collected at 400-568 nm.

3.5 Experimental procedures for selectivity screen towards $O_2^{\bullet-}$ (Figure 3a)

Blank: Probe **LW-OTf** (2.4 µM) was diluted to 1 mL with PBS buffer (10 mM, pH 7.4).

$O_2^{\bullet-}$: $O_2^{\bullet-}$ (18 µM) was added to **LW-OTf** in DMSO, and after 1 min the mixture was diluted to 1 mL with PBS buffer (10 mM, pH 7.4) and incubated for 10 min. The final concentration of **LW-OTf** in PBS buffer solution (with 1% DMSO) was 2.4 µM.

Other ROS/RNS or metal ions: The appropriate ROS/RNS or metal ion was added to **LW-OTf** in DMSO, and after 1 min the mixture was diluted to 1 mL with PBS buffer (10 mM, pH 7.4) and incubated for 10 min. The final concentration of **LW-OTf** in PBS buffer solution (with 1% DMSO) was 2.4 µM.

3.6 Experimental of pH titrations of LW-OTf with $O_2^{\bullet-}$ (Figure S3)

Blank: Probe **LW-OTf** (2.4 µM) was diluted to 1 mL with PBS buffer (10 mM) at various pH's and the mixtures were incubated for 10 min.

$O_2^{\bullet-}$: $O_2^{\bullet-}$ (18 µM) was added to **LW-OTf** in DMSO, and after 1 min the mixture was diluted to 1 mL with PBS buffer (10 mM) at various pH's and incubated for 10 min. The final concentration of **LW-OTf** in PBS buffer solution (with 1% DMSO) was 2.4 µM.

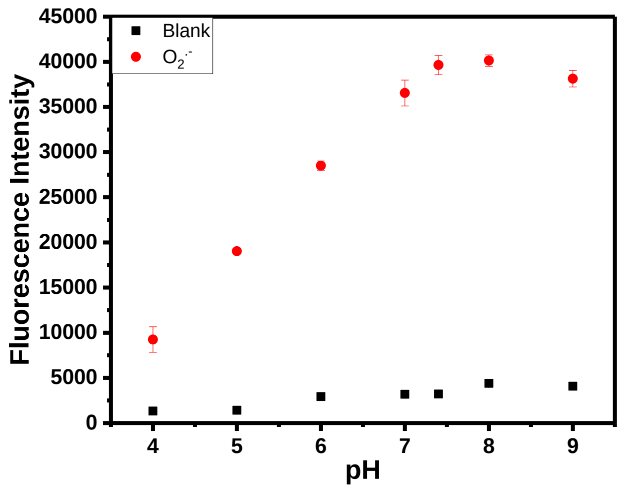

Figure S3. Fluorescence spectra of **LW-OTf** (2.4 µM, black squares) only and after the addition of $O_2^{\bullet-}$ (18 µM, red circles) at various pH's. $\lambda_{ex/em}$ = 675/710 nm.

3.7 Time-course fluorescence measurements of LW-OTf and $O_2^{\bullet-}$ (Figure S4).

Blank: Probe **LW-OTf** (2.4 µM) was diluted to 1 mL with PBS buffer (10 mM, pH 7.4, 1% DMSO). 200 µL were transferred into a microcuvette and fluorescence measurements were performed immediately.

$O_2^{\bullet-}$: $O_2^{\bullet-}$ (18 µM) was added to **LW-OTf** in DMSO, and after 1 min, the mixture was diluted to 1 mL with PBS buffer (10 mM, pH 7.4). 200 µL were transferred into a microcuvette and fluorescence measurements were performed immediately. The final concentration of **LW-OTf** in PBS buffer solution (with 1% DMSO) was 2.4 µM.

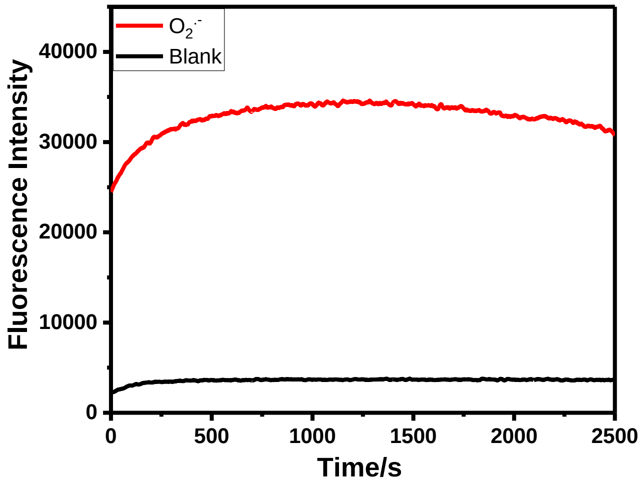

Figure S4. Time course of changes in the fluorescence of probe **LW-OTf** (2.4 µM, black curve), and probe **LW-OTf** (2.4 µM) followed by the addition of $O_2^{\bullet-}$ (18 µM, red curve). $\lambda_{ex/em}$ = 675/710 nm.

3.8 Experimental procedures for the selectivity screen towards $ONOO^-$ (Figure 3b)

Blank: $O_2^{\bullet -}$ (5 μM) was added to **LW-OTf** in DMSO, and after 1 min the mixture was diluted to 1 mL with PBS buffer (10 mM, pH 7.4) and incubated for 7 min. The final concentration of **LW-OTf** in PBS buffer solution (with 1% DMSO) was 2.4 μM.

$ONOO^-$: $O_2^{\bullet -}$ (5 μM) was added to **LW-OTf** in DMSO, and after 1 min $ONOO^-$ (4.2 μM) was added to the solution. After an additional 1 min, the mixture was diluted to 1 mL with PBS buffer (10 mM, pH 7.4) and incubated for 7 min. The final concentration of **LW-OTf** in PBS buffer solution (with 1% DMSO) was 2.4 μM.

Other ROS/RNS or metal ions: $O_2^{\bullet -}$ (5 μM) was added to **LW-OTf** in DMSO, and after 1 min. the appropriate ROS/RNS or metal ion was added. After an additional 1 min, the mixture was diluted to 1 mL with PBS buffer (10 mM, pH 7.4) and incubated for 7 min. The final concentration of **LW-OTf** in PBS buffer solution (with 1% DMSO) was 2.4 μM.

3.9 Experimental procedures for pH titrations of LW-OTf with $ONOO^-$ (Figure S5)

Blank: $O_2^{\bullet -}$ (5 μM) was added to **LW-OTf** in DMSO, and after 1 min the mixture was diluted to 1 mL with PBS buffer a various pH's (10 mM) and incubated for 7 min. The final concentration of **LW-OTf** in PBS buffer solution (with 1% DMSO) was 2.4 μM.

$ONOO^-$: $O_2^{\bullet -}$ (5 μM) was added **to LW-OTf** in DMSO, and after 1 min $ONOO^-$ (4.2 μM) was added. After an additional 1 min, the mixture was was diluted to 1 mL with PBS buffer a various pH's (10 mM) and incubated for 7 min. The final concentration of **LW-OTf** in PBS buffer solution (with 1% DMSO) was 2.4 μM.

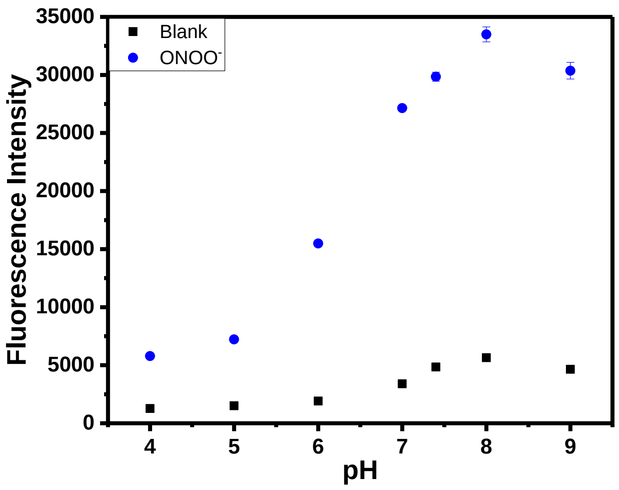

Figure S5. Fluorescence spectra of probe **LW-OTf** (2.4 μM) with $O_2^{\bullet -}$ (5 μM) (black squares), and **LW-OTf** (2.4 μM) with $O_2^{\bullet -}$ (5 μM) followed by the addition of $ONOO^-$ (4.2 μM) (blue circles) at various pH's. $\lambda_{ex/em}$ = 360/461 nm.

3.10 Time-course fluorescence measurements of LW-OTf with $ONOO^-$ (Figure S6).

Blank: $O_2^{\bullet -}$ (5 μM) was added to **LW-OTf** in DMSO, and after 1 min the mixture was diluted to 1 mL with PBS buffer (10 mM, pH 7.4). The final concentration of **LW-OTf** in PBS buffer solution (with 1% DMSO) was 2.4 μM. 200 μL were transferred into a microcuvette, and fluorescence measurements were performed immediately.

$ONOO^-$: $O_2^{\bullet -}$ (5 μM) was added to **LW-OTf** in DMSO, and after 1 min $ONOO^-$ (4.2 μM) was added. After an additional 1 min, the mixture was diluted to 1 mL with PBS buffer (10 mM, pH 7.4). The final

concentration of **LW-OTf** in PBS buffer solution (with 1% DMSO) was 2.4 μM. 200 μL were transferred into a microcuvette, and fluorescence measurements were performed immediately.

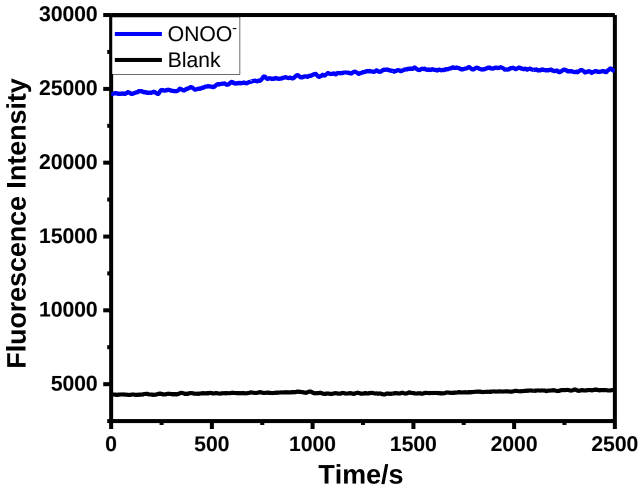

Figure S6. Time course of changes for the fluorescence of probe **LW-OTf** (2.4 μM) with $O_2^{\bullet-}$ (5 μM), and **LW-OTf** (2.4 μM) with $O_2^{\bullet-}$ (5 μM) followed by the addition of $ONOO^-$ (4.2 μM). $\lambda_{ex/em}$ = 360/461 nm.

4. Mass spectroscopic analysis

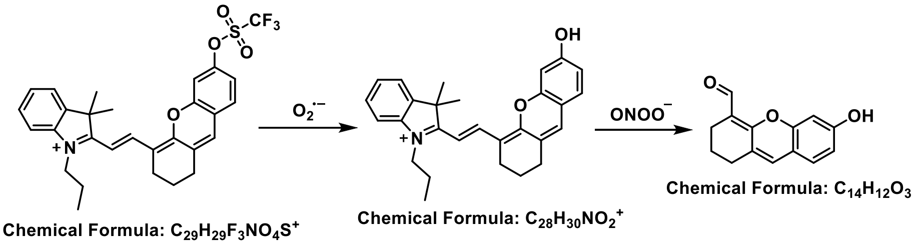

Scheme S1. Proposed mechanism for the reaction of probe **LW-OTf** upon sequential additions of $O_2^{\bullet-}$ then $ONOO^-$.

Probe **LW-OTf** (4.8 mM, in DMSO) was diluted to 19 μM in methanol (**Figure S7**), then $O_2^{\bullet-}$ (60 μM) was added (**Figure S8**), followed by addition of $ONOO^-$ (20 μM) (**Figure S9** and **S10**).

Walkup MS Report

**Data File**	xt-01-002_Pos_LoopInjection_MS_09610.d	**Sample Name**	xt-01-002
**Sample Type**	Sample	**Position**	P1-A2
**Instrument Name**	6545 QTof	**User Name**	Xue Tian
**Acq Method**	Pos_LoopInjection_MS.m	**Acquired Time**	08/01/2020 15:39:45
**IRM Calibration Status**	Success	**DA Method**	Pos_LoopInjection_MS.m
**Comment**			

**Sample Group**		**Info.**	
**Walkup Sample Description**		**Walkup Method**	Pos_LoopInjection_MS
**Formula**	C29H29F3NO4S	**Walkup Method Description**	Positive mode ionization using loop injection with common organic molecule isotope model
**Stream Name**	LC 1	**Acquisition SW Version**	6200 series TOF/6500 series Q-TOF B.09.00 (B9044.0)

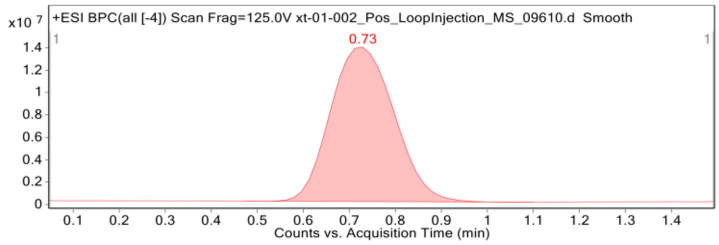

Figure 1: Base peak chromatogram

User Chromatogram Peak List

RT (min)	Area	Area %	Area Sum (%)	Base Peak (m/z)	Width (min)
0.73	132989282	100.00	100.00	544.1741	0.180

Compound Table

Compound Label	RT (min)	Observed mass (m/z)	Neutral observed mass (Da)	Theoretical mass (Da)	Mass error (ppm)	Isotope match score (%)
Cpd 1: C29 H29 F3 N O4 S	0.73	544.1774	544.1779	544.1769	1.77	97.73

Mass errors of between -5.00 and 5.00 ppm with isotope match scores above 60% are considered confirmation of molecular formulae

Walkup MS Report

Compound specific information

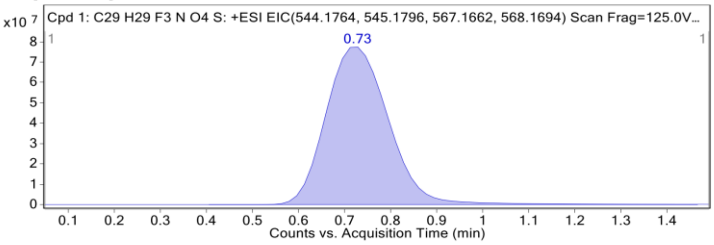

Figure: Extracted ion chromatogram (EIC) of compound.

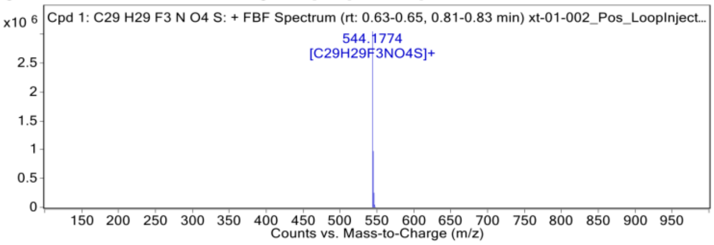

Figure: Full range view of Compound spectra and potential adducts.

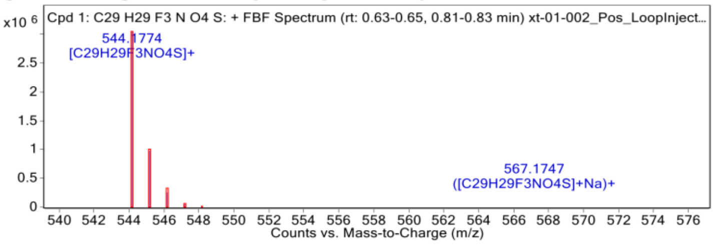

Figure: Zoomed Compound spectra view
(red boxes indicating expected theoretical isotope spacing and abundance)

Compound isotope peak List

m/z	z	Abund	Formula	Ion
544.1774	1	3054717.8	C29H29F3NO4S	M+
545.1803	1	978029.8	C29H29F3NO4S	M+
546.1801	1	255355.3	C29H29F3NO4S	M+
547.1797	1	49570.5	C29H29F3NO4S	M+
548.1798	1	7626.0	C29H29F3NO4S	M+
567.1747	1	189.7	C29H29F3NO4S	(M+Na)+

--- End Of Report ---

Page 2 of 2 Printed at: 15:26 on:10/01/2020

Figure S7. HRMS of probe **LW-OTf**.

Walkup MS Report

**Data File**	NIR + superoxide_Pos_5mins_MS_09611.d	**Sample Name**	NIR + superoxide
**Sample Type**	Sample	**Position**	P1-A6
**Instrument Name**	6545 QTof	**User Name**	Xue Tian
**Acq Method**	Pos_5mins_MS.m	**Acquired Time**	08/01/2020 15:46:48
**IRM Calibration Status**	Success	**DA Method**	Pos_5mins_MS.m
**Comment**			

**Sample Group**		**Info.**	
**Walkup Sample Description**		**Walkup Method**	Pos_5Mins_C18
**Formula**	C28H30NO2	**Walkup Method Description**	Positive mode ionization using C18 column chromatography
**Stream Name**	LC 1	**Acquisition SW Version**	6200 series TOF/6500 series Q-TOF B.09.00 (B9044.0)

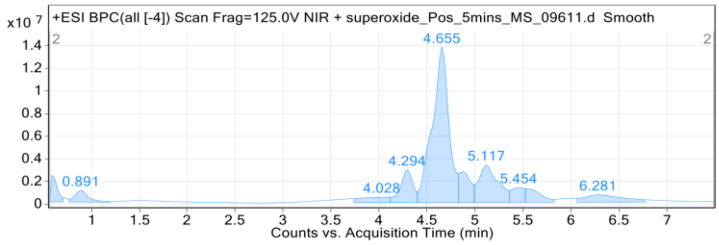

Figure 1: Base peak chromatogram

User Chromatogram Peak List

RT (min)	Area	Area %	Area Sum (%)	Base Peak (m/z)	Width (min)
0.60	10817414	6.51	3.21	157.0350	0.069
0.89	10376469	6.25	3.08	474.2636	0.143
4.03	10220236	6.15	3.03	149.9527	0.351
4.29	26005300	15.65	7.71	185.1151	0.139
4.66	166135869	100.00	49.28	544.1895	0.174
4.87	22898445	13.78	6.79	743.3154	0.124
5.12	46293935	27.87	13.73	755.6421	0.189
5.45	12413818	7.47	3.68	441.3003	0.138
5.59	13459580	8.10	3.99	441.3003	0.204
6.28	18499034	11.13	5.49	172.9779	0.400

Compound Table

Compound Label	RT (min)	Observed mass (m/z)	Neutral observed mass (Da)	Theoretical mass (Da)	Mass error (ppm)	Isotope match score (%)
Cpd 1: C28 H30 N O2	4.52	412.2283	412.2288	412.2277	2.67	96.45

Mass errors of between -5.00 and 5.00 ppm with isotope match scores above 60% are considered confirmation of molecular formulae

Walkup MS Report

UNIVERSITY OF BATH

Compound specific information

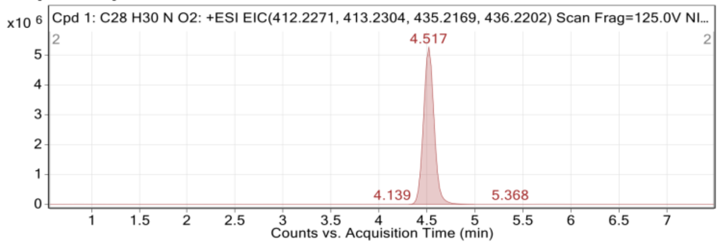

Figure: Extracted ion chromatogram (EIC) of compound.

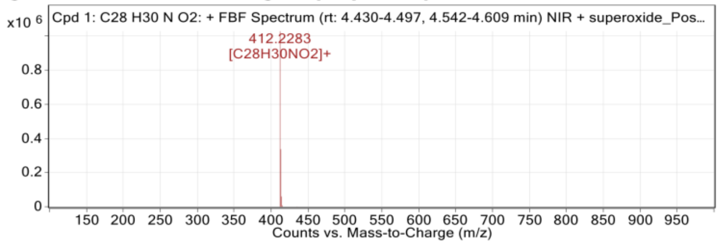

Figure: Full range view of Compound spectra and potential adducts.

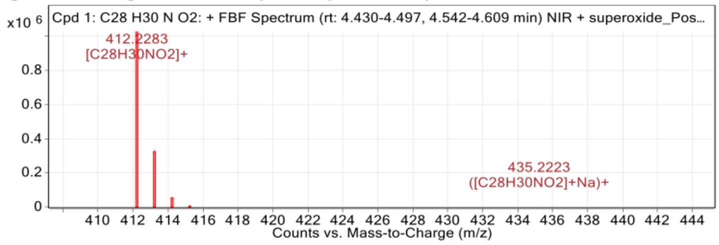

Figure: Zoomed Compound spectra view
(red boxes indicating expected theoretical isotope spacing and abundance)

Compound isotope peak List

m/z	z	Abund	Formula	Ion
412.2283	1	1028296.3	C28H30NO2	M+
413.2314	1	336874.9	C28H30NO2	M+
414.2340	1	59780.6	C28H30NO2	M+
415.2355	1	8099.3	C28H30NO2	M+
435.2223	1	1014.9	C28H30NO2	(M+Na)+

--- End Of Report ---

Figure S8. LC-MS of **LW-OTf** + $O_2^{\bullet -}$, showing cleavage of **LW-OTf** to the related phenol.

Walkup MS Report

**Data File**	nir +superoxide+onoo1_Pos_5mins_MS_09624.d	**Sample Name**	nir +superoxide+onoo1
**Sample Type**	Sample	**Position**	P1-A5
**Instrument Name**	6545 QTof	**User Name**	Xue Tian
**Acq Method**	Pos_5mins_MS.m	**Acquired Time**	1/9/2020 5:06:45 PM
**IRM Calibration Status**	Success	**DA Method**	Pos_5mins_MS.m
**Comment**			

**Sample Group**		**Info.**	
**Walkup Sample Description**		**Walkup Method**	Pos_5Mins_C18
**Formula**	C14H12O3	**Walkup Method Description**	Positive mode ionization using C18 column chromatography
**Stream Name**	LC 1	**Acquisition SW Version**	6200 series TOF/6500 series Q-TOF B.09.00 (B9044.0)

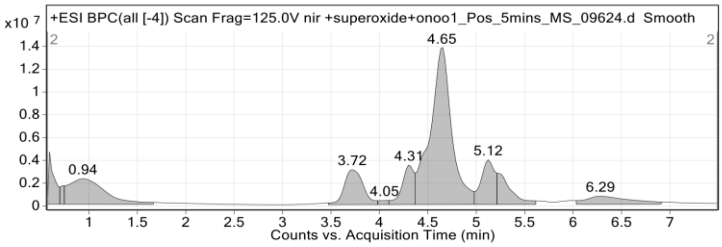

Figure 1: Base peak chromatogram

User Chromatogram Peak List

RT (min)	Area	Area %	Area Sum (%)	Base Peak (m/z)	Width (min)
0.60	18347084	8.56	4.00	101.0029	0.060
0.73	4450464	2.08	0.97	137.0015	0.050
0.94	59453480	27.74	12.96	137.0015	0.400
3.72	41951353	19.57	9.14	185.1147	0.220
4.05	2459372	1.15	0.54	149.9527	0.090
4.31	29348047	13.69	6.40	185.1150	0.130
4.65	214326951	100.00	46.71	544.1857	0.220
5.12	38189350	17.82	8.32	755.6376	0.140
5.26	27300386	12.74	5.95	938.7991	0.190
6.29	23042531	10.75	5.02	172.9774	0.460

Compound Table

Compound Label	RT (min)	Observed mass (m/z)	Neutral observed mass (Da)	Theoretical mass (Da)	Mass error (ppm)	Isotope match score (%)
Cpd 1: C14 H12 O3	4.52	229.0860	228.0779	228.0786	-3.44	99.11

Mass errors of between -5.00 and 5.00 ppm with isotope match scores above 60% are considered confirmation of molecular formulae

Walkup MS Report

UNIVERSITY OF BATH

Compound specific information

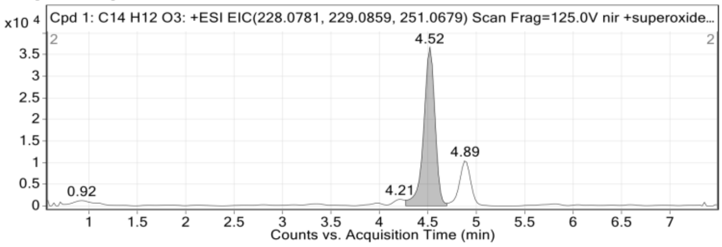

Figure: Extracted ion chromatogram (EIC) of compound.

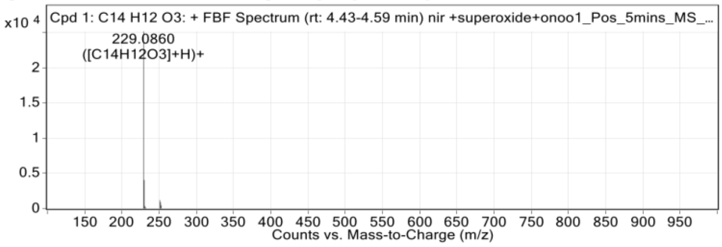

Figure: Full range view of Compound spectra and potential adducts.

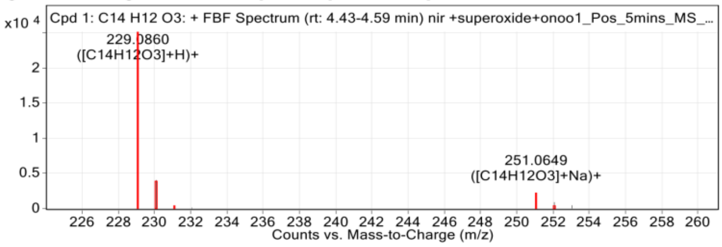

Figure: Zoomed Compound spectra view
(red boxes indicating expected theoretical isotope spacing and abundance)

Compound isotope peak List

m/z	z	Abund	Formula	Ion
229.0860	1	25117.6	C14H12O3	(M+H)+
230.0898	1	4054.7	C14H12O3	(M+H)+
231.0954	1	351.0	C14H12O3	(M+H)+
232.0744	1	128.1	C14H12O3	(M+H)+
251.0649	1	1199.3	C14H12O3	(M+Na)+
252.0618	1	905.2	C14H12O3	(M+Na)+
253.0413	1	458.2	C14H12O3	(M+Na)+

--- End Of Report ---

Figure S9. LC-MS of **LW-OTf** + $O_2^{\bullet -}$ + $ONOO^-$, showing cleavage of **LW-OTf** to the related phenol and C=C linker oxidative cleavage.

Walkup MS Report

**Data File**	nir+superoxide+onoo_Pos_5mins_MS_09688.d	**Sample Name**	nir+superoxide+onoo
**Sample Type**	Sample	**Position**	P1-A2
**Instrument Name**	6545 QTof	**User Name**	Xue Tian
**Acq Method**	Pos_5mins_MS.m	**Acquired Time**	1/13/2020 3:15:29 PM
**IRM Calibration Status**	Success	**DA Method**	Pos_5mins_MS.m
**Comment**			

**Sample Group**		**Info.**	
**Walkup Sample Description**		**Walkup Method**	Pos_5Mins_C18
**Formula**	C13H17NO	**Walkup Method Description**	Positive mode ionization using C18 column chromatography
**Stream Name**	LC 1	**Acquisition SW Version**	6200 series TOF/6500 series Q-TOF B.09.00 (B9044.0)

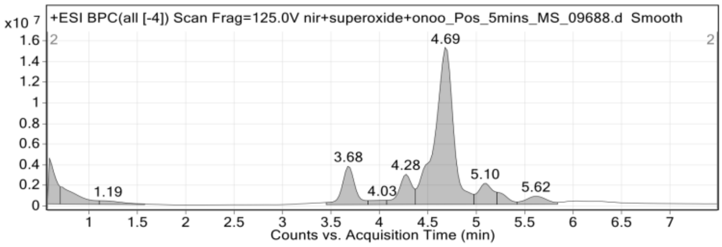

Figure 1: Base peak chromatogram

User Chromatogram Peak List

RT (min)	Area	Area %	Area Sum (%)	Base Peak (m/z)	Width (min)
0.60	21955206	10.64	6.03	101.0036	0.080
0.73	20448328	9.91	5.62	101.0036	0.210
1.19	5875997	2.85	1.61	157.0359	0.270
3.68	34861773	16.90	9.58	202.1604	0.140
4.03	4706336	2.28	1.29	149.9537	0.160
4.28	27626349	13.39	7.59	185.1162	0.140
4.69	206283422	100.00	56.67	544.1897	0.200
5.10	21689635	10.51	5.96	755.6424	0.150
5.28	8612793	4.18	2.37	562.1901	0.140
5.62	11965674	5.80	3.29	419.3192	0.230

Compound Table

Compound Label	RT (min)	Observed mass (m/z)	Neutral observed mass (Da)	Theoretical mass (Da)	Mass error (ppm)	Isotope match score (%)
Cpd 1: C13 H17 N O	4.73	204.1388	203.1314	203.1310	1.95	97.95

Mass errors of between -5.00 and 5.00 ppm with isotope match scores above 60% are considered confirmation of molecular formulae

Walkup MS Report

Compound specific information

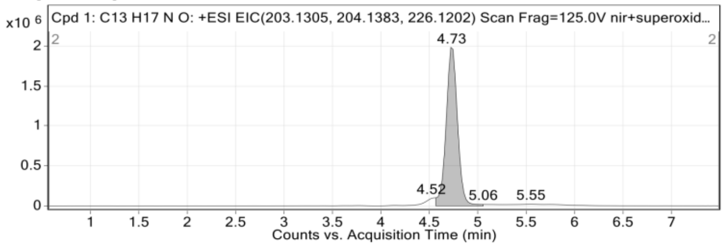

Figure: Extracted ion chromatogram (EIC) of compound.

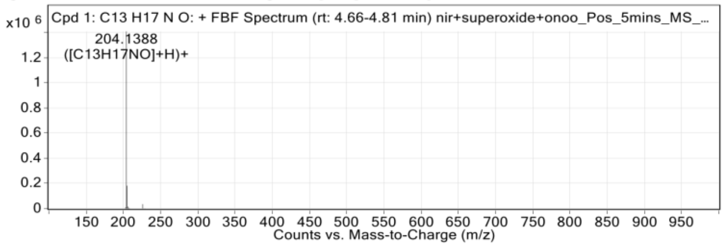

Figure: Full range view of Compound spectra and potential adducts.

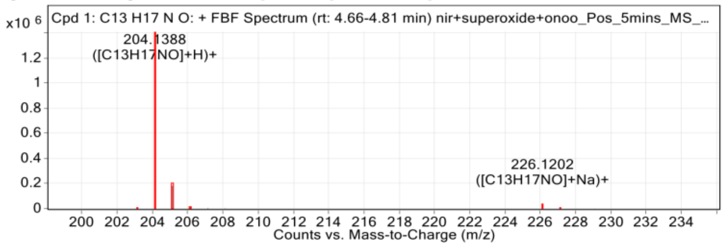

Figure: Zoomed Compound spectra view
(red boxes indicating expected theoretical isotope spacing and abundance)

Compound isotope peak List

m/z	z	Abund	Formula	Ion
203.1285	1	9304.6	C13H17NO	M+
204.1388	1	1408518.3	C13H17NO	(M+H)+
205.1421	1	181691.7	C13H17NO	(M+H)+
206.1455	1	14867.7	C13H17NO	(M+H)+
207.1379	1	4869.3	C13H17NO	(M+H)+
208.1345	1	2689.3	C13H17NO	(M+H)+
226.1202	1	35028.0	C13H17NO	(M+Na)+
227.0918	1	420.7	C13H17NO	(M+Na)+
228.0908	1	279.7	C13H17NO	(M+Na)+
229.0700	1	919.8	C13H17NO	(M+Na)+

--- End Of Report ---

Page 2 of 2 Printed at: 3:24 PM on:1/13/2020

Figure S10. LC-MS of **LW-OTf** + $O_2^{\bullet-}$ + $ONOO^-$, showing C=C linker oxidative cleavage.

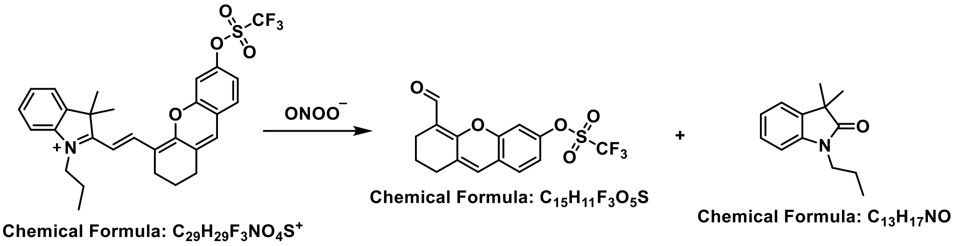

Scheme S2. Proposed mechanism for the reaction of **LW-OTf** with $ONOO^-$.

Probe **LW-OTf** (4.8 mM, in DMSO) was diluted to 19 μM in methanol (**Figure S11**), then $ONOO^-$ (95 μM) was added (**Figure S12**).

Walkup MS Report

**Data File**	nirsendor 20um_Pos_LoopInjection_MS_11354.d	**Sample Name**	nirsendor 20um
**Sample Type**	Sample	**Position**	P1-B4
**Instrument Name**	6545 QTof	**User Name**	Xue Tian
**Acq Method**	Pos_LoopInjection_MS.m	**Acquired Time**	06/08/2020 14:58:53
**IRM Calibration Status**	Success	**DA Method**	Pos_LoopInjection_MS.m
**Comment**			

**Sample Group**		**Info.**	
**Walkup Sample Description**		**Walkup Method**	Pos_LoopInjection_MS
**Formula**	C29H29F3NO4S	**Walkup Method Description**	Positive mode ionization using loop injection with common organic molecule isotope model
**Stream Name**	LC 1	**Acquisition SW Version**	6200 series TOF/6500 series Q-TOF B.09.00 (B9044.0)

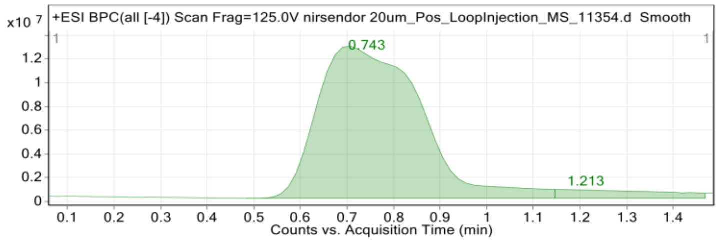

Figure 1: Base peak chromatogram

User Chromatogram Peak List

RT (min)	Area	Area %	Area Sum (%)	Base Peak (m/z)	Width (min)
0.74	198418075	100.00	94.51	544.1732	0.253
1.21	11523971	5.81	5.49	101.0013	0.277

Compound Table

Compound Label	RT (min)	Observed mass (m/z)	Neutral observed mass (Da)	Theoretical mass (Da)	Mass error (ppm)	Isotope match score (%)
Cpd 1: C29 H29 F3 N O4 S	0.74	544.1778	544.1783	544.1769	2.58	97.92

Mass errors of between -5.00 and 5.00 ppm with isotope match scores above 60% are considered confirmation of molecular formulae

Walkup MS Report

Compound specific information

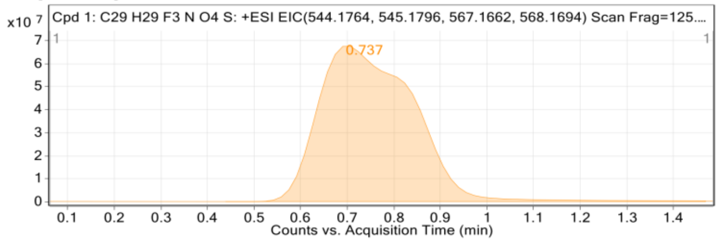

Figure: Extracted ion chromatogram (EIC) of compound.

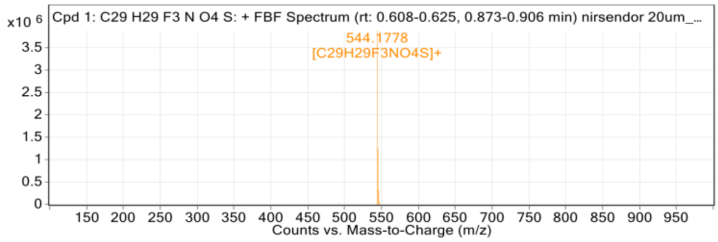

Figure: Full range view of Compound spectra and potential adducts.

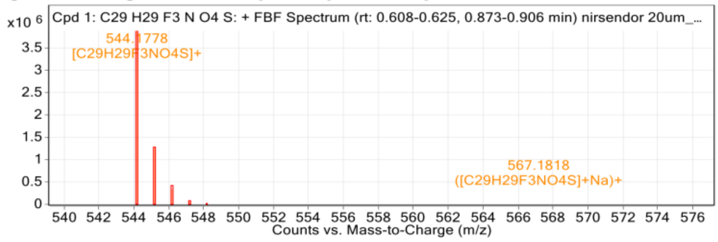

Figure: Zoomed Compound spectra view
(red boxes indicating expected theoretical isotope spacing and abundance)

Compound isotope peak List

m/z	z	Abund	Formula	Ion
544.1778	1	3872682.0	C29H29F3NO4S	M+
545.1807	1	1260751.9	C29H29F3NO4S	M+
546.1804	1	331803.7	C29H29F3NO4S	M+
547.1800	1	62182.9	C29H29F3NO4S	M+
548.1807	1	9099.0	C29H29F3NO4S	M+
567.1818	1	669.5	C29H29F3NO4S	(M+Na)+
568.1759	1	493.6	C29H29F3NO4S	(M+Na)+

--- End Of Report ---

Page 2 of 2 Printed at: 11:13 on:27/08/2020

Figure S11. HRMS of probe **LW-OTf**.

Walkup MS Report

**Data File**	SENSOR+ 5eq ONOO_Pos_5mins_MS_11357.d	**Sample Name**	SENSOR+ 5eq ONOO
**Sample Type**	Sample	**Position**	P1-B7
**Instrument Name**	6545 QTof	**User Name**	Xue Tian
**Acq Method**	Pos_5mins_MS.m	**Acquired Time**	06/08/2020 15:22:05
**IRM Calibration Status**	Success	**DA Method**	Pos_5mins_MS.m
**Comment**			

**Sample Group**		**Info.**	
**Walkup Sample Description**		**Walkup Method**	Pos_5Mins_C18
**Formula**	C15H11F3O5S,C13H17NO	**Walkup Method Description**	Positive mode ionization using C18 column chromatography
**Stream Name**	LC 1	**Acquisition SW Version**	6200 series TOF/6500 series Q-TOF B.09.00 (B9044.0)

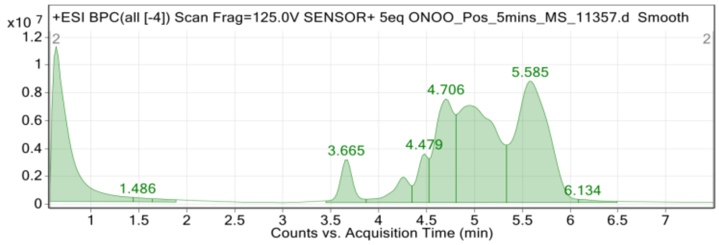

Figure 1: Base peak chromatogram

User Chromatogram Peak List

RT (min)	Area	Area %	Area Sum (%)	Base Peak (m/z)	Width (min)
0.64	136225768	64.63	18.67	101.0032	0.204
1.49	3315875	1.57	0.45	101.0026	0.155
1.73	2990271	1.42	0.41	101.0025	0.195
3.67	28491148	13.52	3.91	202.1585	0.133
4.26	24169334	11.47	3.31	185.1144	0.186
4.48	27440884	13.02	3.76	592.1960	0.119
4.71	101452535	48.13	13.91	594.1761	0.206
4.95	191240558	90.73	26.21	441.2970	0.387
5.59	210783150	100.00	28.89	419.3155	0.389
6.13	3427251	1.63	0.47	149.9523	0.223

Compound Table

Compound Label	RT (min)	Observed mass (m/z)	Neutral observed mass (Da)	Theoretical mass (Da)	Mass error (ppm)	Isotope match score (%)
Cpd 1: C15 H11 F3 O5 S	4.90	361.0357	360.0284	360.0279	1.34	98.73
Cpd 2: C13 H17 N O	3.81	204.1379	203.1307	203.1310	-1.35	86.45

Mass errors of between -5.00 and 5.00 ppm with isotope match scores above 60% are considered confirmation of molecular formulae

Walkup MS Report

Compound specific information

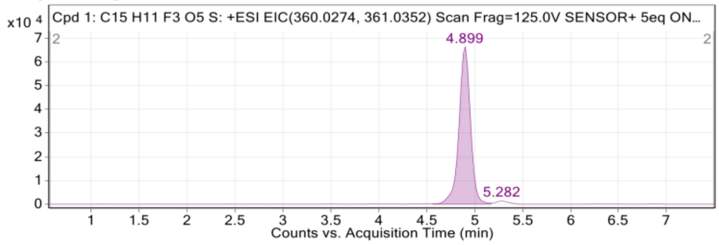

Figure: Extracted ion chromatogram (EIC) of compound.

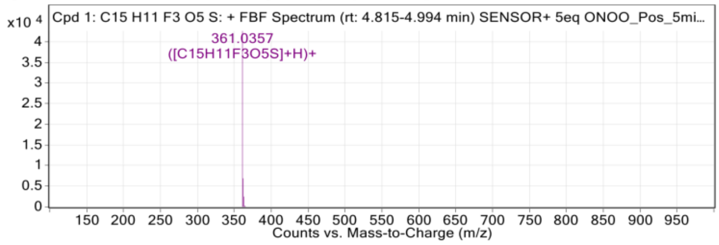

Figure: Full range view of Compound spectra and potential adducts.

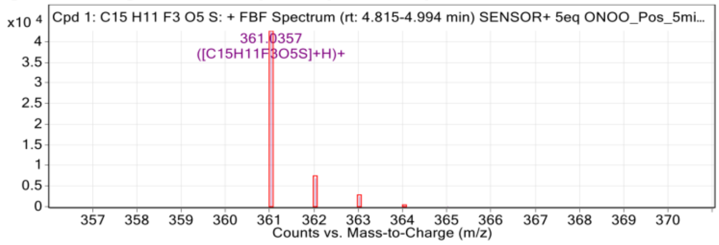

Figure: Zoomed Compound spectra view
(red boxes indicating expected theoretical isotope spacing and abundance)

Compound isotope peak List

m/z	z	Abund	Formula	Ion
361.0357	1	42634.1	C15H11F3O5S	(M+H)+
362.0388	1	6906.0	C15H11F3O5S	(M+H)+
363.0348	1	2473.9	C15H11F3O5S	(M+H)+
364.0365	1	296.7	C15H11F3O5S	(M+H)+
365.0313	1	113.6	C15H11F3O5S	(M+H)+

Walkup MS Report

Compound specific information

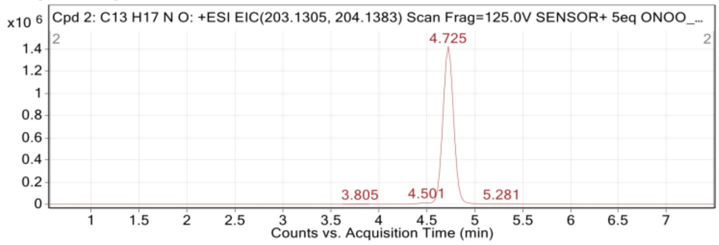

Figure: Extracted ion chromatogram (EIC) of compound.

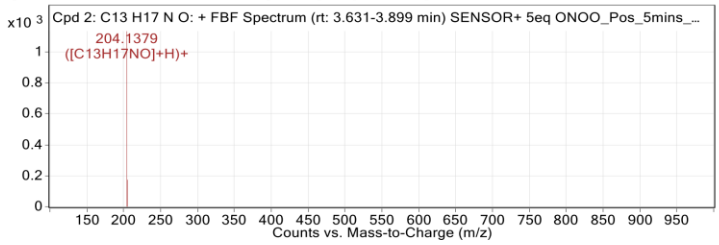

Figure: Full range view of Compound spectra and potential adducts.

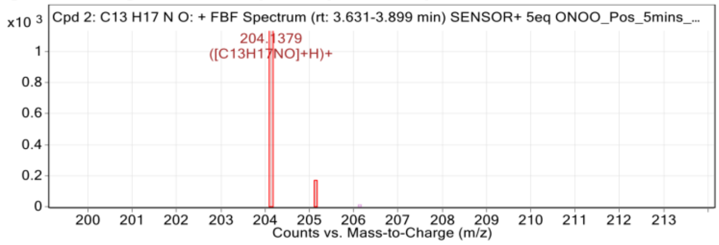

Figure: Zoomed Compound spectra view
(red boxes indicating expected theoretical isotope spacing and abundance)

Compound isotope peak List

m/z	z	Abund	Formula	Ion
204.1379	1	1134.5	C13H17NO	(M+H)+
205.1417	1	173.9	C13H17NO	(M+H)+

--- End Of Report ---

Figure S12. LC-MS of **LW-OTf** + ONOO$^-$, showing C=C linker oxidative cleavage.

5. Protocols for cell culture

Human hepatocytes (HL-7702) were purchased from the Cell Bank of the Chinese Academy of Sciences (Shanghai, China). Cells were cultured in high-glucose DMEM supplemented with 10 % fetal bovine serum, 1 % penicillin and 1 % streptomycin (w v^{-1}) at 37 °C in a 5 % CO_2/95 % air MCO-15AC incubator (SANYO, Tokyo, Japan).

6. Fluorescence imaging in live cells and MTT assay

6.1 MTT assay.

3-(4,5-dimethylthiazol-2-yl)-2,5-diphenyltetrazolium bromide (MTT) assays were carried out to evaluate the toxicity of probe **LW-OTf**. Hepatocytes (10^6 cells mL^{-1}) were seeded into 96-well microtiter plates with total volumes of 200 μL $well^{-1}$. After 12 h of incubation, various concentrations of **LW-OTf** (0 M, 1×10^{-3} M, 1×10^{-4} M, 1×10^{-5} M, 1×10^{-6} M, 1×10^{-7} M, 1×10^{-8} M and 1×10^{-9} M) were added, and the cells were cultured for another 24 h. Subsequently, an MTT solution (20 μL, 5 mg mL^{-1}, in DMEM) was added to each well. After 4 h, the MTT solution was removed, and DMSO (150 μL) was added to each well. Finally, the absorbance at 490 nm was measured using a Triturus microplate reader.

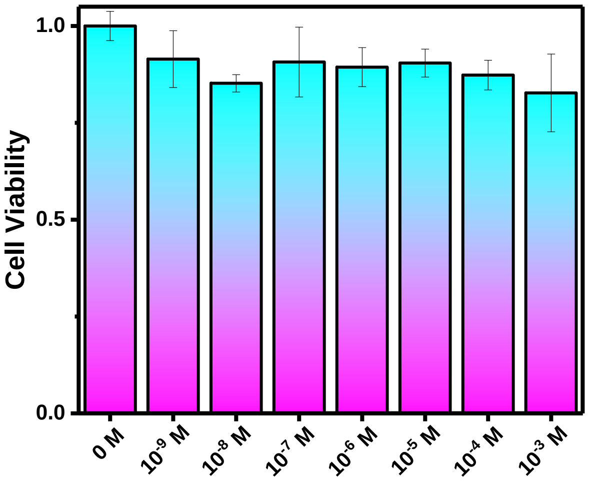

Figure S13. Cell toxicity of **LW-OTf** towards HL-7702 cells with an incubation time of 24 h. Error bars represent s.d.

6.2 Fluorescence imaging experiments with cells.

Living cells were detached and reseeded onto 15 mm glass-bottomed dishes 24 h before confocal imaging and two-photon (TP) fluorescence imaging. For **Figure S14-S17**, confocal imaging was performed on Leica SP8 high-resolution fluorescence microscope with red channel (λ_{ex}=633 nm and λ_{em}=680-780 nm) and blue channel (λ_{ex}=405 nm and λ_{em}=410-500 nm). For **Figure 4**, two-photon (TP) fluorescence images were obtained with a Zeiss 880 NLO microscope and the supporting software ZEN 2 lite. TP images of probe fluorescence were obtained with NIRF red channel (λ_{ex} = 633 nm and λ_{em}=635-747 nm) and TPEF blue channel (λ_{ex} = 720 nm and λ_{em}=420-550 nm). For data analysis, the average fluorescence intensity per image for each set of experimental conditions was obtained by selecting the regions of interest. Each experiment was repeated five separate times with identical results. All data are expressed as the mean ± S.D.

6.2.1 Fluorescence imaging of HL-7702 cells (pre-incubated with Tiron) and treatment with either 2-ME or 2-ME/SIN-1.

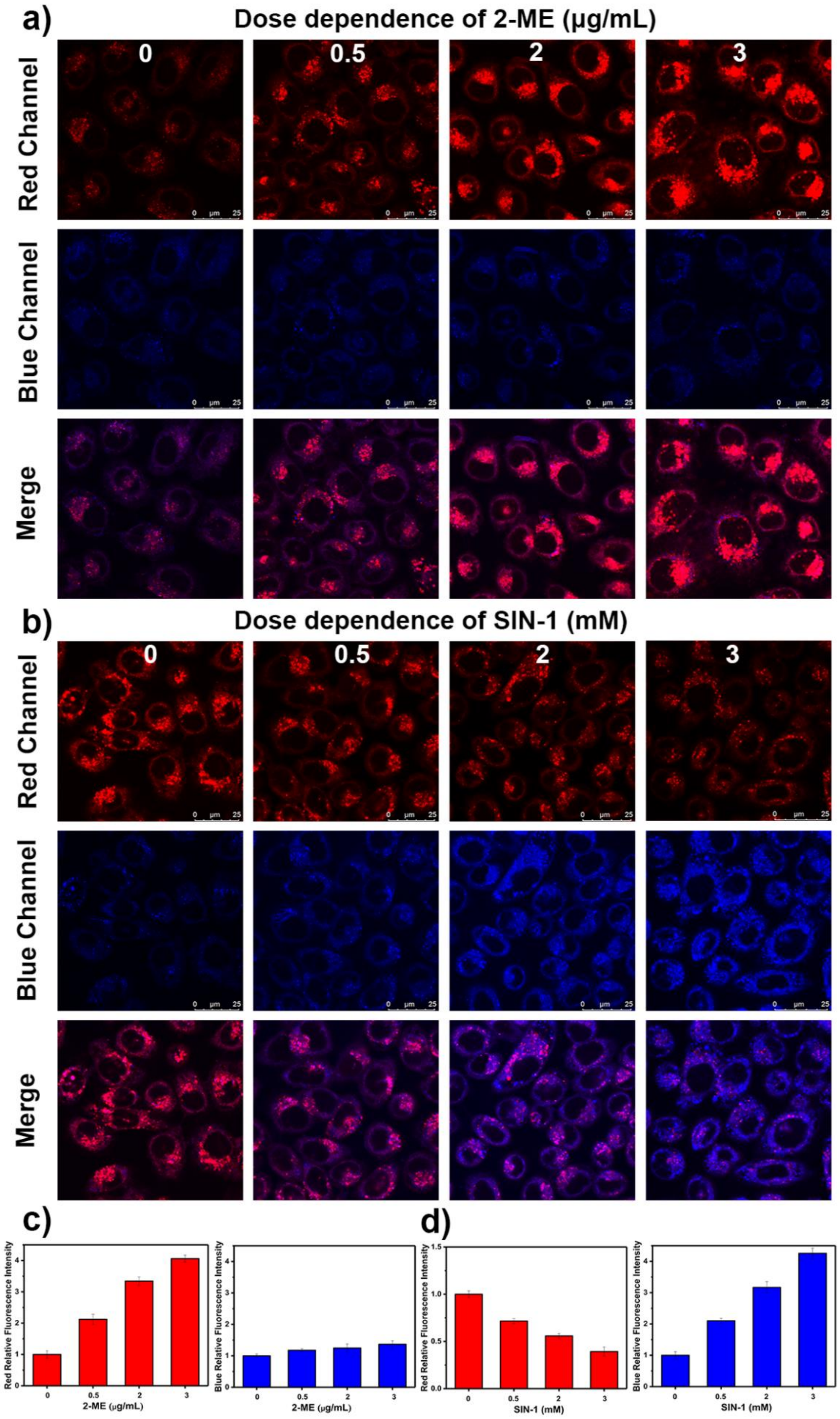

Figure S14. Confocal imaging of $O_2^{\bullet-}$/ONOO$^-$ levels in hepatocytes. (a) HL-7702 cells were pretreated with Tiron (10 μM) for 0.5 h, with various concentrations of 2-ME (0, 0.5, 2.0 and 3.0 μg/mL) for 0.5 h, and then exposed to probe **LW-OTf** (2.4 μM) for 15 min. (b) HL-7702 cells were pretreated with Tiron (10 μM) for 0.5 h,

incubated with 2-ME (3.0 μg/mL) for 0.5 h, exposed to probe **LW-OTf** (2.4 μM) for 15 min, and finally incubated with various concentrations of SIN-1 (0, 0.5, 2.0, and 3.0 mM) for another 0.5 h. (a, b) Red fluorescence channel for $O_2^{\bullet-}$: λ_{ex} = 633 nm, λ_{em} = 680-780 nm; blue fluorescence channel for $ONOO^-$: λ_{ex} = 405 nm, λ_{em} = 410-500 nm. (c) Red and blue relative fluorescence intensity output with various doses of 2-ME in (a). The fluorescence intensity of the control group (2-ME, 0 μg/mL) is defined as 1.0. (d) Relative fluorescence intensity output with various doses of SIN-1 in (b) in the red and blue channels. The fluorescence intensity of the control group (SIN-1, 0 mM) is defined as 1.0. Note: The data are expressed as the mean ± SD. Results were confirmed in quintuplicate. Blue channels using excitation at 405 nm represent one-photon imaging.

6.2.2 Fluorescence imaging of APAP-induced injury in HL-7702 cells.

Experimental procedure for Figure 4 and Figure S15

HL-7702 cells were divided into three groups. The control group cells were stained with probe **LW-OTf** (2.4 μM) for 15 min. The model group cells were incubated with APAP (20 mM) for 1 h, then stained with probe **LW-OTf** (2.4 μM) for 15 min. To investigate injury remediation, HL-7702 cells were pretreated with BHA (500 μM) for 1 h and then incubated with APAP (20 mM) for 1 h, followed by staining with probe **LW-OTf** (2.4 μM) for 15 min. The cell culture medium of each group was removed, and all cells were washed with 1.0 mL of PBS three times before fluorescence imaging. For **Figure 4**, two-photon photographs were taken imaged by Zeiss 880 NLO microscope and the supporting software ZEN 2 lite. For **Figure S15**, one-photon confocal photographs were taken imaged by Leica SP8 high-resolution fluorescence microscope and Leica Application Suite X software.

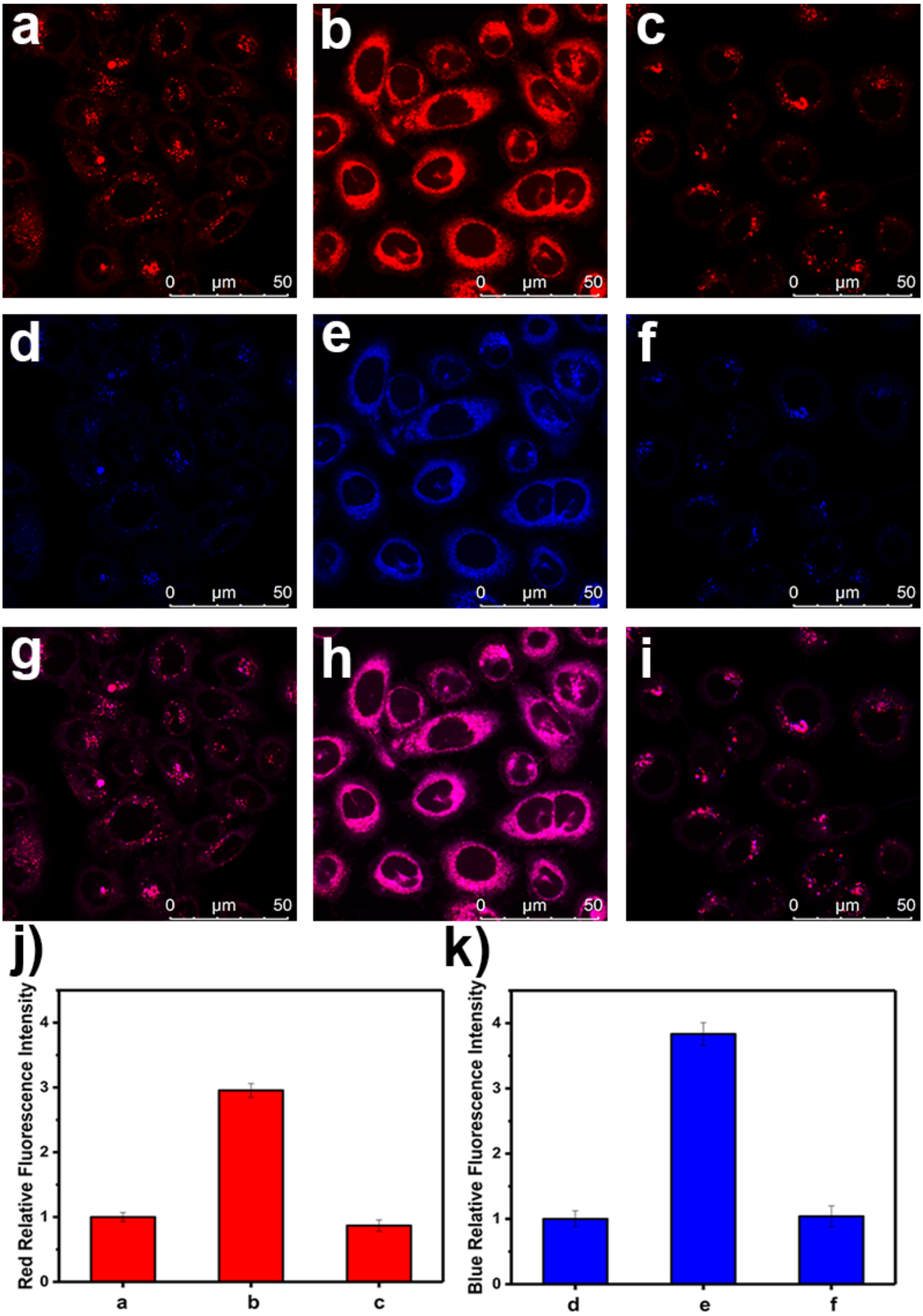

Figure S15. Confocal images of APAP-induced injury with HL-7702 cells. (a, d and g) Cells were stained with probe **LW-OTf** (2.4 μM) for 15 min. (b, e, and h) Cells were incubated with APAP (20 mM) for 1 h, and then stained with probe **LW-OTf** (2.4 μM) for 15 min. (c, f and i) Cells were pretreated with BHA (500 μM) for 1 h and then incubated with APAP (20 mM) for 1 h, followed by staining with probe **LW-OTf** (2.4 μM) for another 15 min. (a-c) Red fluorescence channel for $O_2^{\bullet-}$: λ_{ex} = 633 nm, λ_{em} = 680-780 nm; (d-f) Blue fluorescence channel for $ONOO^-$: λ_{ex} = 405 nm, λ_{em} = 410-500 nm. (g-i) Merged fluorescence channel. (j) Red relative fluorescence intensity output of a-c. (k) Blue relative fluorescence intensity output of d-f. Note: the fluorescence intensity of control group is defined as 1.0. The data are expressed as the mean ± SD. Comparable results were obtained in five independent experiments. Blue channels using excitation 405 nm represent one-photon imaging.

6.2.3 Co-localization assay of LW-OTf.

Experimental procedure for Figure S16. HL-7702 cells were incubated with 2-ME (2.0 μg/mL) for 0.5 h and then co-incubated with probe **LW-OTf** (2.4 μM) and each corresponding commercial organelle-specific dye for 15 min.

Experimental procedure for Figure S17. HL-7702 cells were co-incubated with 2-ME (2.0 μg/mL) and SIN-1 (2.0 mM) for 0.5h, followed by staining with probe **LW-OTf** (2.4 μM) and each corresponding commercial organelle-specific dye for 15 min.

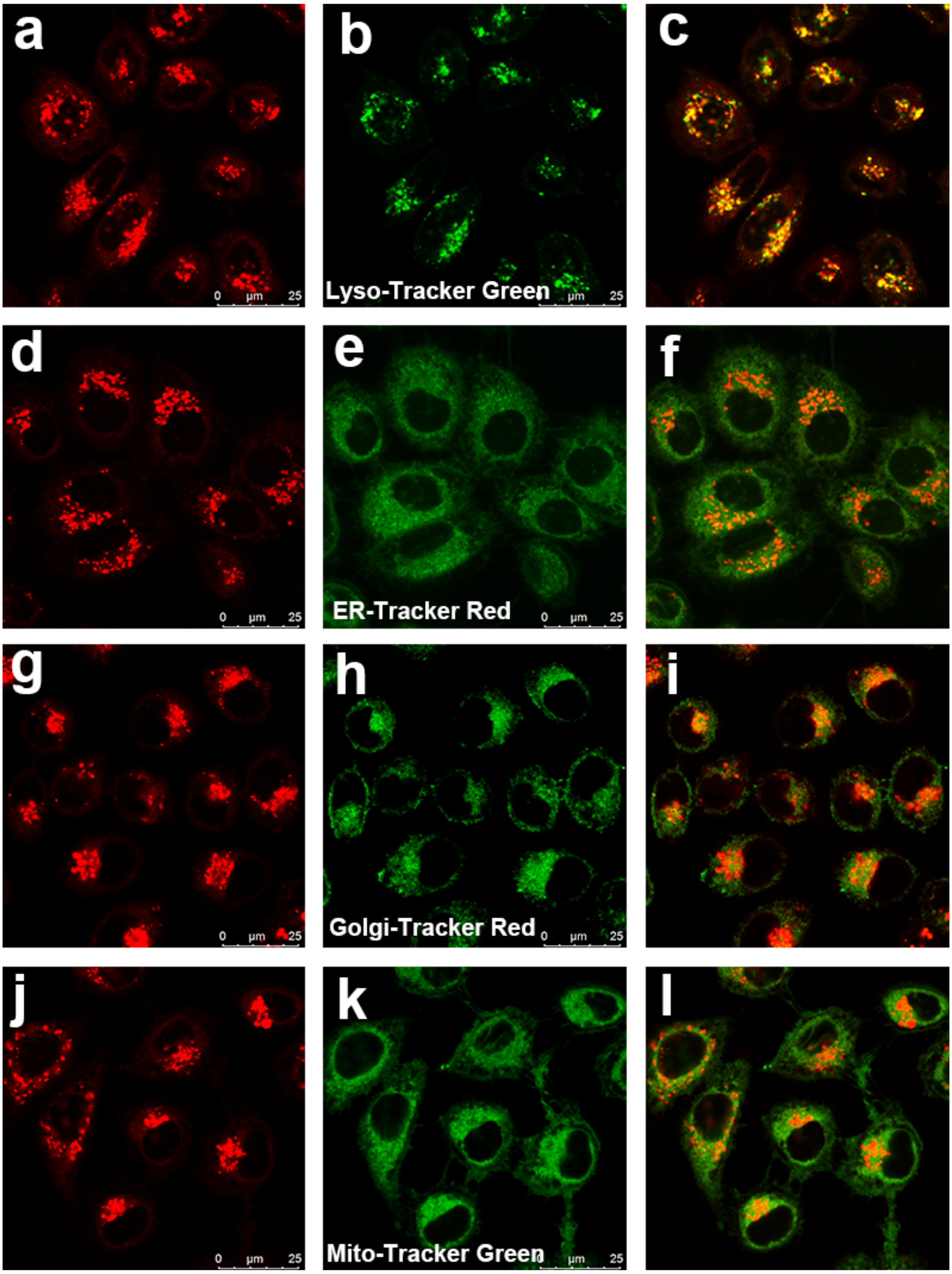

Figure S16. Confocal images of HL-7702 cells co-stained with probe **LW-OTf** and the corresponding commercial organelle-specific dyes. (a, d, g, and j) Fluorescence image of probe **LW-OTf** (2.4 μM, red channel, Ex = 633 nm, collected 680-780 nm) in cells pretreated with 2-ME (2.0 μg/mL) for 0.5h. (b) Fluorescence image of Lyso-Tracker Green (75 nM, green channel, Ex = 488 nm, collected 495-550 nm). (e) Fluorescence image of ER-Tracker Red (75 nM, green channel, Ex = 561 nm, collected 600-630 nm). (h) Fluorescence image of Golgi-Tracker Red (75 nM, green channel, Ex = 561 nm, collected 600-630 nm). (k) Fluorescence image of Mito-Tracker Green (60 nM, green channel, Ex = 488 nm, collected 495-550 nm). (c), (f), (i), (l) Overlay of (a) and (b), (d) and (e), (g) and (h), (j) and (k), respectively. Pearson's correlation of lysosomes, endoplasmic reticulum, Golgi apparatus and mitochondria are 0.8659, 0.5132, 0.3931 and 0.3452, respectively.

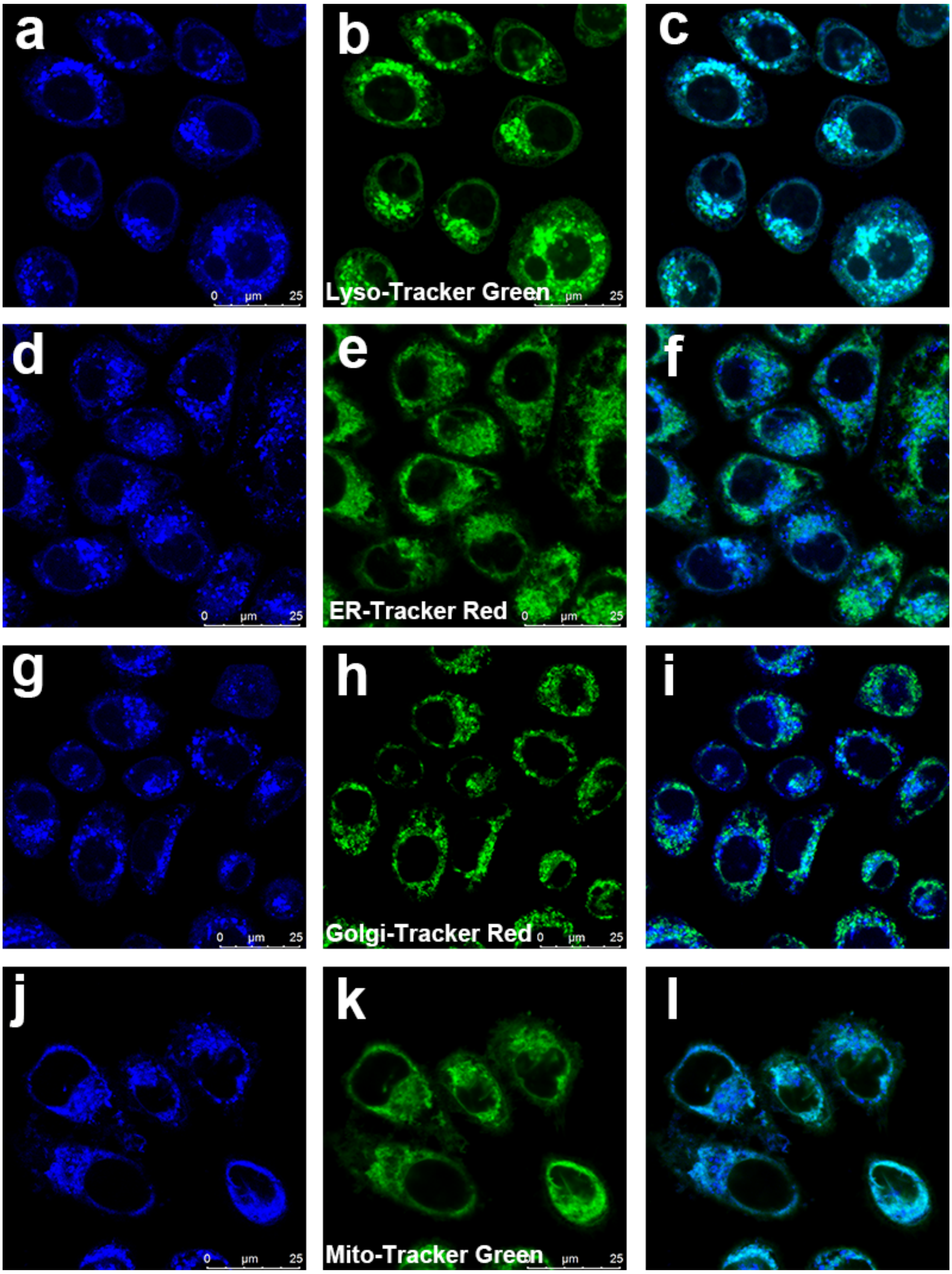

Figure S17. Confocal images of HL-7702 cells co-stained with probe **LW-OTf** and the corresponding commercial organelle-specific dyes. (a, d, g, and j) Fluorescence image of probe (2.4 μM, blue channel, Ex = 405 nm, collected 410-480 nm) in cells co-incubated with 2-ME (2.0 μg/mL) and SIN-1 (2.0 mM) for 0.5h. (b) Fluorescence image of Lyso-Tracker Green (75 nM, green channel, Ex = 488 nm, collected 495-550 nm). (e) Fluorescence image of ER-Tracker Red (75 nM, green channel, Ex = 561 nm, collected 600-630 nm). (h) Fluorescence image of Golgi-Tracker Red (75 nM, green channel, Ex = 561 nm, collected 600-630 nm). (k) Fluorescence image of Mito-Tracker Green (60 nM, green channel, Ex = 488 nm, collected 495-550 nm). (c), (f), (i), (l) Overlay of (a) and (b), (d) and (e), (g) and (h), (j) and (k), respectively. Pearson's correlation of lysosomes, endoplasmic reticulum, Golgi apparatus and mitochondria are 0.8962, 0.2902, 0.4155 and 0.4752, respectively. Note: blue channels using excitation 405 nm represent one-photon imaging.

7. Near-infrared fluorescence imaging of APAP-induced injury in mice

Four to six-week old male C57 mice were purchased from the laboratory animal center of Shandong University (Jinan, China). Permission for the animal experiments was obtained from the Shandong Normal University authorities.

Experimental procedure for Figure 5a and 5b
For *in vivo* imaging of the livers of mice with drug-induced liver injury the model group mice were intraperitoneally injected with acetaminophen APAP, (400 mg/kg and 600 mg/kg). The control group were given APAP in analgesic dosage (200 mg/kg). After 6 h, all mice were first intraperitoneally injected with anaesthetic (10% chloral hydrate, 100 μL), and 10 min later with probe **LW-OTf** (200 μL, 48 μM), and left for 15 minutes. All mice underwent surgical treatment to expose the liver for *in vivo* imaging. The images were recorded in the red channel ($\lambda_{ex/em}$ = 660/710 nm).

Experimental procedure for Figure 5c and 5d
To evaluate the deep tissue penetration of probe **LW-OTf**, the model group mice were intraperitoneally injected with acetaminophen APAP, (400 mg/kg and 600 mg/kg). The control group were given APAP in analgesic dosage (200 mg/kg). After 6 h, the mice were first intraperitoneally injected with anaesthetic (10% chloral hydrate, 100 μL), and 10 min later with **LW-OTf** (200 μL, 48 μM), and left for 15 min before depilation. The images were recorded in the red channel ($\lambda_{ex/em}$ = 660/710 nm).

Experimental procedure for Figure 5e and 5f
The mice were divided into three groups. The first group received an intraperitoneal injection of APAP (200 mg/kg). The second group was given an intraperitoneal injection of APAP (600 mg/kg). The third group was first given an intraperitoneal injection of 400 mg/kg NAC, and 1 h later was injected intraperitoneally with 600 mg/kg APAP. All three groups were injected peritoneally with **LW-OTf** (200 μL, 48 μM) after administering APAP, after intervals of 30 min, 2h and 6h The images were recorded in the red channel ($\lambda_{ex/em}$ = 660/710 nm).

Experimental procedure for Figure 7a and 7b
The model group mice were intraperitoneally injected with APAP (600 mg/kg) for 6 h. The control group were given the same volume of saline (100 μL). Subsequently, all the mice were intraperitoneally injected anaesthetic (10% chloral hydrate, 100 μL) and left for 10 min. After intraperitoneal injection of **LW-OTf** (200 μL, 48 μM) and 15 min incubation the mice were killed and major organs were excised, collected and imaged in the red channel ($\lambda_{ex/em}$ = 660/710 nm).

8. Two-photon fluorescence imaging of APAP-induced injury in mice

Experimental procedure for Figure 6
Four to six-week old male C57 mice were fasted for 12 h and divided into three groups. The model group mice were intraperitoneally injected with APAP (400 mg/kg) and APAP (600 mg/kg) for 6 h, respectively. The control group were given APAP (200 mg/kg). Subsequently, all the mice were intraperitoneally injected with anaesthetic (10% chloral hydrate, 100 μL), and 10 min later with probe **LW-OTf** (200 μL, 48 μM). After 15 minutes, the mice underwent surgical treatment to expose the liver for two-photon fluorescence imaging. The liver was imaged with excitation at 720 nm and the emission was collected between 397−571 nm. Two-photon 3D imaging was acquired with the z-stack mode of the microscope.

9. Hematoxylin and eosin (H&E) staining of main organs in mice

Experimental procedure for Figure 7
The model group of mice were injected intraperitoneally with APAP (600 mg/kg) for 6 h. The control group were

given the same volume of physiological saline. Subsequently, the mice were intraperitoneally injected with anaesthetic (10% chloral hydrate, 100 μL), wait 10 min. Then the mice were injected intraperitoneally with **LW-OTf** (200 μL, 48 μM) and left for 15 min. Subsequently, all the mice were dissected to isolate the spleen, lungs, heart, kideys and liver. After imaging in the red channel, the isolated organs were fixed in 4 % paraformaldehyde for tissue staining. The samples were dehydrated, embedded, sectioned and stained by hematoxylin and eosin. The sections were imaged with optical microscope (NIKON Eclipse ci) and the supporting software (NIKON digital sight DS-FI2, MADE IN JAPAN).

Beginning with splenic tissue cells (Figure 7c and 7h), red and white pulp boundaries were clear, with a large quantitity of white pulp, and visible cord and sinus of the red pulp, abundant lymphocytes, and normal overall structure and morphology with no obvious necrosis or bleeding, indicating no damage to the spleen. Similarly, the pulmonary tissue cells of both control and model groups (Figure 7d and 7i) appeared healthy, as indicated by the tightly arranged bronchiolar epithelial cells with no obvious degeneration, necrosis, or distinct secretions in the cavity. Moreover, the alveolar walls were thin with no significant thickening and their structure was complete; no obvious inflammatory cell infiltration was observed in pulmonary interstitium or alveoli. Cardiac myocytes of the control and model groups (Figure 7e and 7j) also appeared healthy, staining uniformly. The myocardial fibers were regularly and tightly arranged, and with no obvious degeneration or necrosis, and no obvious inflammatory cell infiltration. Renal tissue cells in both groups (Figure 7f and 7k) showed no abnormal morphology and structure of the glomerular tissues and the renal tubules were tightly arranged. No obvious degeneration, necrosis or brush border shedding was seen, nor was there any renal interstitial damage, connective tissue hyperplasia, or any obvious inflammatory cell infiltration.

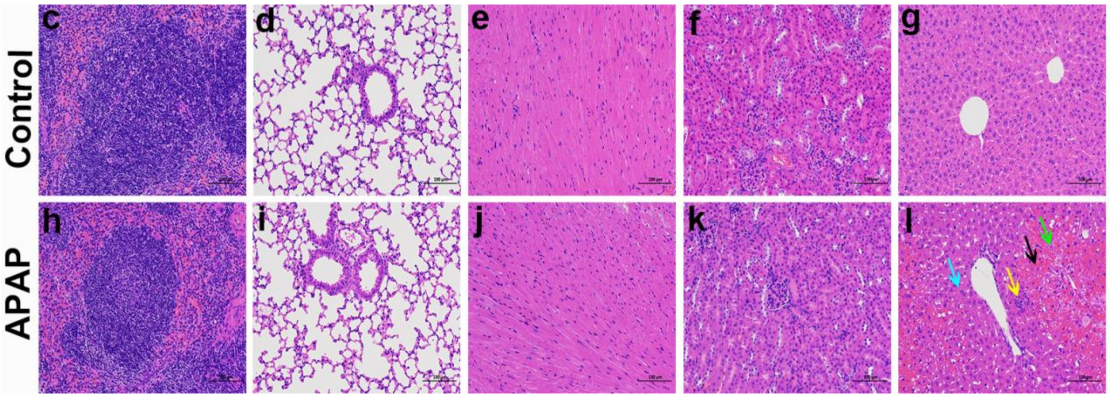

Figure S18. Enlarged hematoxylin and eosin (H&E) staining images of **Figure 7**. (c-l) The hematoxylin and eosin (H&E) staining of main organs in mice. (c-g) Spleen, lung, heart, kidney, liver tissue section of control group. (h-l) Spleen, lung, heart, kidney, liver tissue section of model group with APAP (600 mg/kg)-induced liver injury. (g) The structure of the liver lobules was clear, the hepatocytes were arranged neatly. No obvious degeneration and necrosis of hepatocytes were observed. There was no obvious congestion of hepatic sinusoid. No obvious inflammatory cell infiltration was seen. (l) Compared with the control group, there were obvious liver damages in the model group. Widely congestions and hemorrhages were visible in the sinusoids of the acinar III zone (black arrow). A large amount of hepatocytes steatosis with dense vacuoles in the cytoplasm (blue arrow) were observed. Some hepatocytes with dissolved and disappeared nucleus were necrotic (green arrow). Slight inflammatory infiltration and individual inflammatory foci in the liver lobules could be seen (yellow arrow). Scale bar = 100 μm.

10. Synthesis of probe LW-OTf

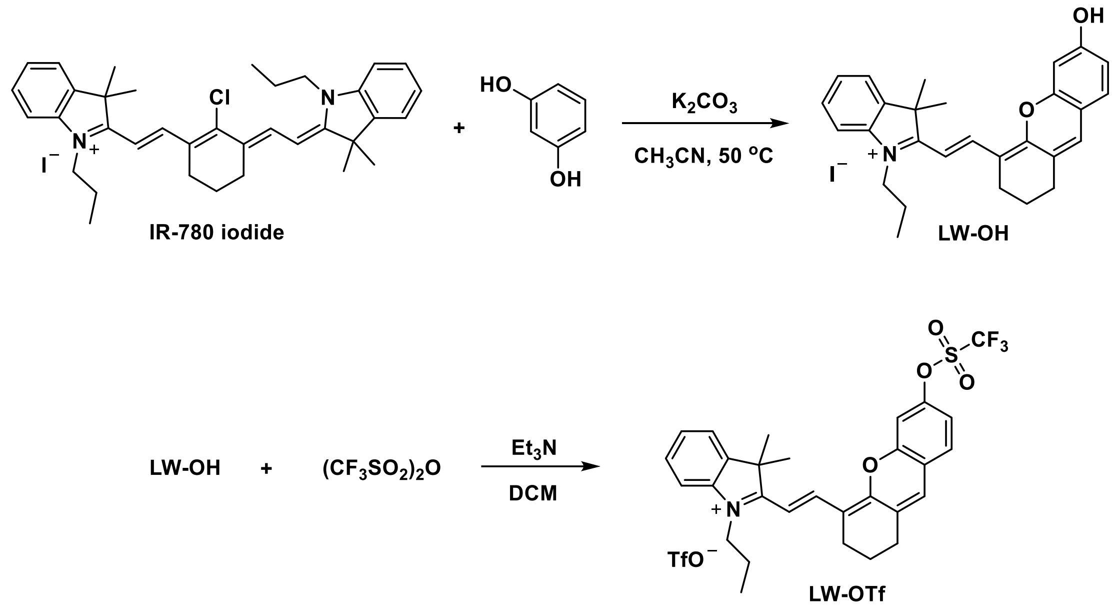

Scheme S3. Synthesis of probe **LW-OTf**.

Synthesis of LW-OH

A mixture of resorcinol (275 mg, 2.5 mmol) and K_2CO_3 (346 mg, 2.5 mmol) in acetonitrile (10.0 mL) was stirred under a nitrogen atmosphere at room temperature for 15 min, before addition of IR-780 iodide (667 mg, 1.0 mmol) in acetonitrile (5.0 mL). The reaction mixture was heated to 50 °C for 2 h. After cooling, the solvent was removed *in vacuo*, and the residue was purified by silica gel chromatography (SiO_2, CH_2Cl_2/MeOH, 80:1 v/v) to afford **LW-OH** as a blue-green solid in 10% yield (56 mg). M.p. 182 °C; ^{1}H NMR (500 MHz, $CDCl_3$) δ_H 11.13 (bs, 1H, -O*H*), 8.63 (d, J = 14.9 Hz, 1H, -N=CC*H*), 7.58-7.15 (m, 8H, Ar*H*), 6.12 (d, J = 14.7 Hz, 1H, -N=CC=C*H*), 4.12 (t, J = 7.8 Hz, 2H, C*H₂*), 2.76 (t, J = 5.9 Hz, 2H, CH_2), 2.66 (t, J = 5.9 Hz, 2H, CH_2), 1.99-1.90 (m, 4H, 2 x CH_2), 1.79 (s, 6H, 2 x -N=CCCH_3), 1.10 (t, J = 7.5 Hz, 3H, -CH_2CH_3); ^{13}C NMR (126 MHz, $CDCl_3$) δ_C δ 176.1, 163.8, 163.4, 157.6, 154.9, 144.9, 141.9, 141.6, 137.0, 129.0, 128.8, 126.6, 125.4, 122.8, 116.9, 114.9, 111.6, 103.2, 101.5, 50.4, 46.8, 29.8, 29.1, 28.7, 21.2, 20.6, 11.9; HRMS (ESI$^+$): calc. for $C_{28}H_{30}NO_2$ $[M-I]^+$ 412.2271 *m/z*, found 412.2271 *m/z*. Characterization data were consistent with previous literature reports.[1]

Synthesis of LW-OTf

To a stirred solution of **LW-OH** (200 mg, 0.37 mmol) in dichloromethane (6.0 mL) was added triethylamine (180 μL, 1.32 mmol). After cooling to 0 °C, triflic anhydride (Tf_2O, 187 μL, 1.11 mmol) was added dropwise, and the reaction was left to stir for 30 min, before warming to room temperature and leaving to stir a further 35 min. The reaction mixture was diluted with water (20 mL) and dichloromethane (30 mL), the layers were separated, and the aqueous phase was extracted with dichloromethane (30 mL) twice more. The combined organics were concentrated to dryness *in vacuo*, and the residue was purified by silica gel column chromatography (SiO_2, CH_2Cl_2/MeOH 60:1, v/v) to afford the title compound **LW-OTf** as a purple solid (178 mg, 69%). M.p. 121 °C; ^{1}H NMR (500 MHz, $CDCl_3$) δ_H 8.61 (d, J = 15.2 Hz, 1H, -N=CC*H*), 7.56 – 7.51 (m, 2H, Ar*H*), 7.50 – 7.42 (m, 3H, Ar*H*), 7.15-7.09 (m, 2H, Ar*H*), 7.03 (s, 1H, Ar*H*), 6.81 (d, J = 15.3 Hz, 1H, -N=CC=C*H*), 4.53 (t, J = 6.9 Hz, 2H, CH_2), 2.78 (t, J = 6.0 Hz, 2H, CH_2), 2.75 (t, J = 6.0 Hz, 2H, CH_2), 2.03-1.91 (m, 4H, 2 x CH_2), 1.82 (s, 6H, 2 x -N=CCCH_3), 1.07 (t, J = 7.4 Hz, 3H, -CH_2CH_3); ^{13}C NMR (126 MHz, $CDCl_3$) δ_C 179.6, 158.4, 153.0, 140.9, 146.4, 142.5, 141.4, 132.2, 129.6, 129.0, 128.9,

128.6, 122.6, 122.2, 120.1 (q, $J_{C\text{-}F}$ = 320.7 Hz, 1C, OTf), 120.1 (q, $J_{C\text{-}F}$ = 320.7 Hz, 1C, OTf), 118.0, 116.2, 113.9, 109.5, 108.0, 51.5, 47.9, 29.6, 28.1, 24.2, 21.8, 20.1, 11.5; ^{19}F NMR (500 MHz, $CDCl_3$) δ_F -72.6 (ArOTf), -78.3 (TfO^-); HRMS (ESI^+): calc. for $C_{29}H_{29}F_3NO_4S$ $[M\text{-}OTf]^+$ 544.1764 *m/z*, found 544.1774 *m/z*.

11. NMR spectra

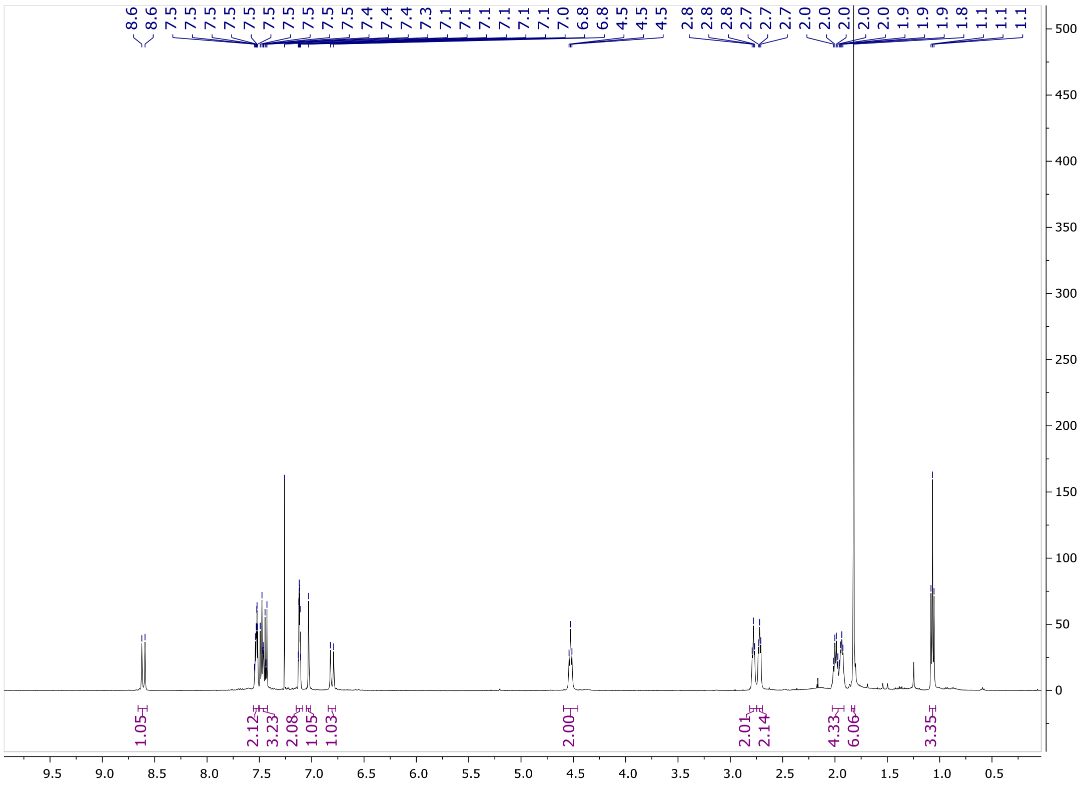

Figure S19. ^{1}H NMR (500 MHz, $CDCl_3$) of **LW-OTf.**

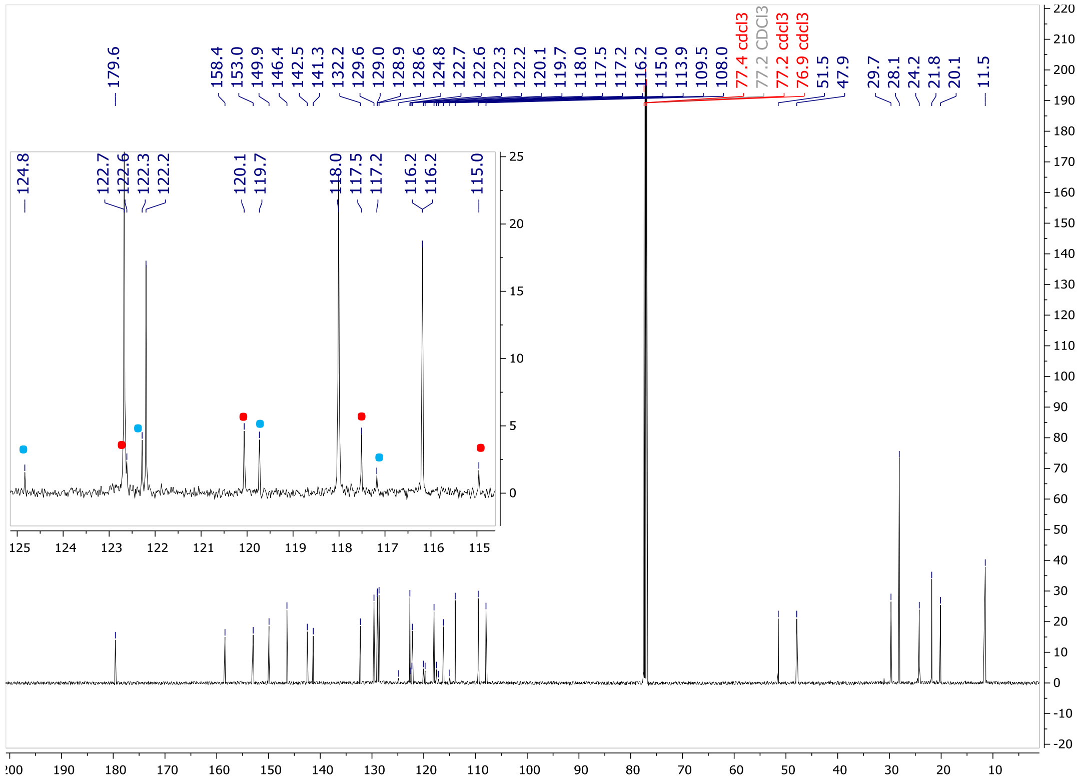

Figure S20. $^{13}C\{^{1}H\}$ NMR (126 MHz, $CDCl_3$) of **LW-OTf**. Inset showing 2 x CF_3 quartets (red and blue are separate quartets).

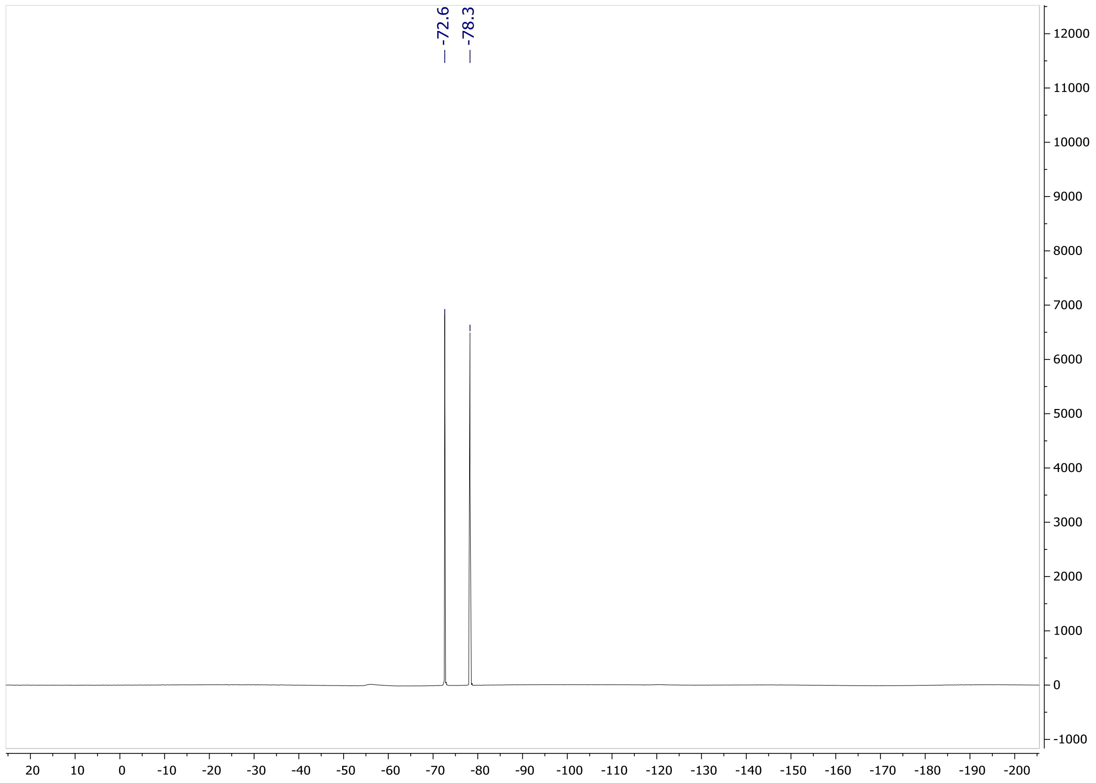

Figure S21. ^{19}F NMR (470 MHz, $CDCl_3$) of **LW-OTf**.

12. Characterisation of LW-OTf triflate salt

Upon characterization of **LW-OTf** following the synthesis described above, it was observed that an anion exchange had occurred, exchanging the original iodide counteranion I^- IR-780 iodide for a triflate TfO^- (by product of the triflation reaction). The experiments described below were therefore carried out in order to confirm this observation.

Two quantitative ^{19}F NMR experiments were carried out initially. The first used a single scan ^{19}F NMR method, producing an inherently quantitative spectrum (**Figure S22**). The second experiment instead used a 16-scan ^{19}F NMR experiment with an increased relaxation time $T1$ = 40 s, allowing for accurate integration of the resulting signals (**Figure S23**). Both experiments showed a 1:1 ratio of the ^{19}F signals, indicating a 1:1 ratio of the two triflate environments, consistent with our suggested structure of salt **LW-OTf**, containing both a phenol triflate moiety and a triflate counteranion.

As it was acknowledged that this equimolar ratio could be coincidental and caused by residual TfOH byproduct from the previous synthetic step, further experiments were carried out (full procedure below). Addition of TfOH to a sample of **LW-OTf** showed an increase in intensity and broadening of the signal at -78.3 ppm by ^{19}F NMR, indicating this peak to be the TfOH/TfO^- signal (**Figure S24**). This mixture was then washed with water, dried, and the resulting product was analyzed by quantitative ^{19}F NMR spectroscopy (**Figures S25 and S26**). This revealed that the intensities of the triflate ^{19}F signals of **LW-OTf** had returned to a 1:1 ratio, indicating that excess triflate had been removed by the wash, and that all remaining signal was in fact part of the complex.

Previous reports in the literature have shown that iodide anions are capable of exchanging with triflates to produce stable heteroatomic salts and quaternary ammonium salts.[2] Additionally, comparison of the pKa's of HI (-9.5 in water, -10.9 in DMSO) and TfOH (-14.7 in water, -14.3 in DMSO) indicates that the former is more likely to protonate and therefore be removed during workup.[3] Moreover, comparison of their boiling points (HI: -35.4 °C; TfOH: 162 °C)[4,5] shows that upon exchange, HI will quickly evaporate even at atmospheric pressure, favoring the formation of the triflate salt. These properties and reports confirm the likelihood of iodide to triflate anion exchange in our hemicyanine system.

At this stage we would like to note that a surprising number of reports of hemicyanine dyes were found in the literature that either overlooked the possibility of anion exchange or ignored the counteranion entirely, likely leading to inaccurate measurements of concentration and stoichiometry. We hope that this thorough characterization of the counter-anion will serve as an example of good practice, hopefully leading to more consistent structural determination and reporting of hemicyanine structures in the future.

Experimental procedure for doping of LW-OTf with TfOH and washing: Triflic acid (5.3 µL, 60 µmol) was added to a stirred solution of **LW-OTf** (20 mg, 29 µmol) in DCM (1.0 mL) at 0 °C. After stirring for 1 h, the solvent was removed *in vacuo*, and quantitative ^{19}F NMR was carried out (**Figures S24**). The product was then re-dissolved in DCM (10 mL), washed with water (3 x 10 mL), dried over $MgSO_4$ and concentrated *in vacuo*. ^{19}F NMR quantitative NMR was then taken (**Figures S25** and **S26**).

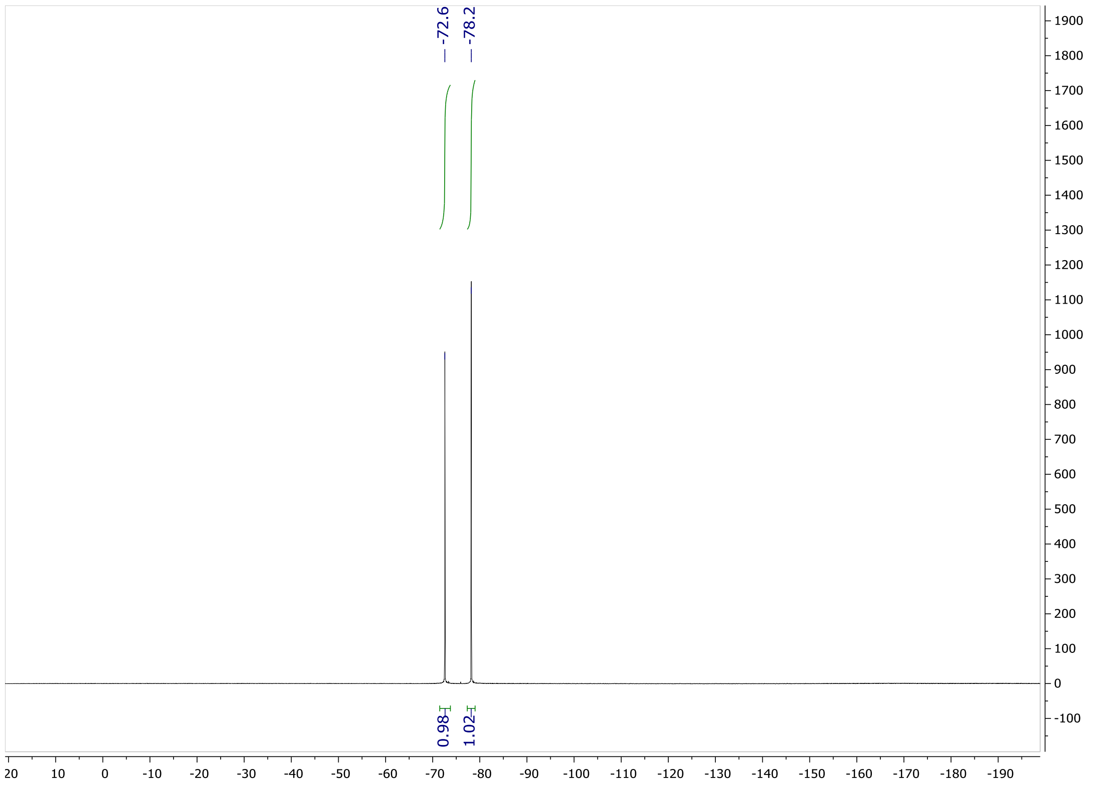

Figure S22. ^{19}F NMR (470 MHz, $CDCl_3$, 1 scan) of **LW-OTf**.

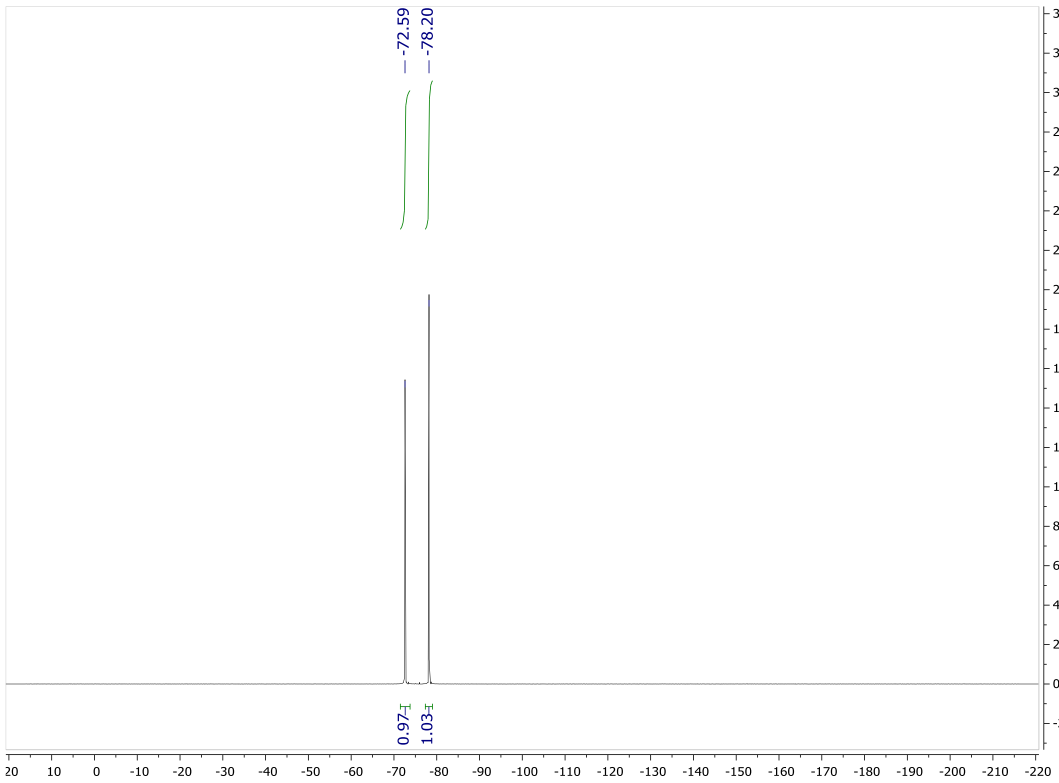

Figure S23. ^{19}F NMR (470 MHz, $CDCl_3$, 16 scans, *T1* = 40 s) of **LW-OTf**.

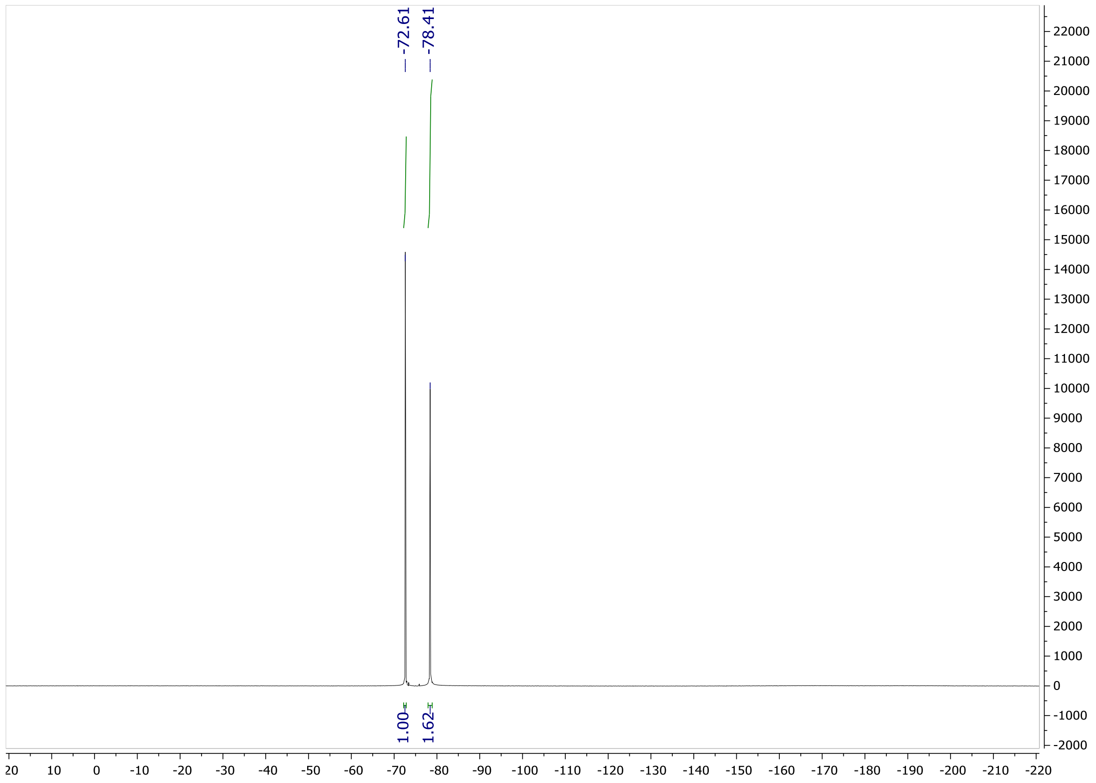

Figure S24. ^{19}F NMR (470 MHz, $CDCl_3$, 16 scans, *T1* = 40 s) of **LW-OTf** + TfOH.

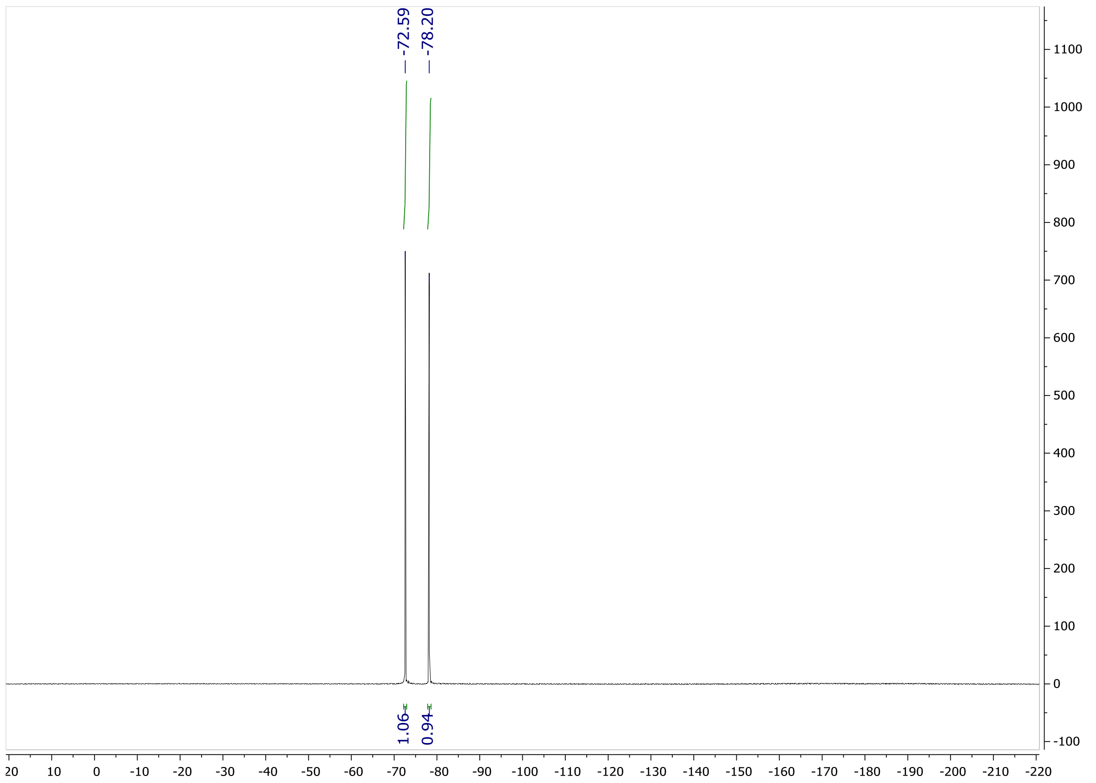

Figure S25. ^{19}F NMR (470 MHz, $CDCl_3$, 1 scan) of washed **LW-OTf** + TfOH.

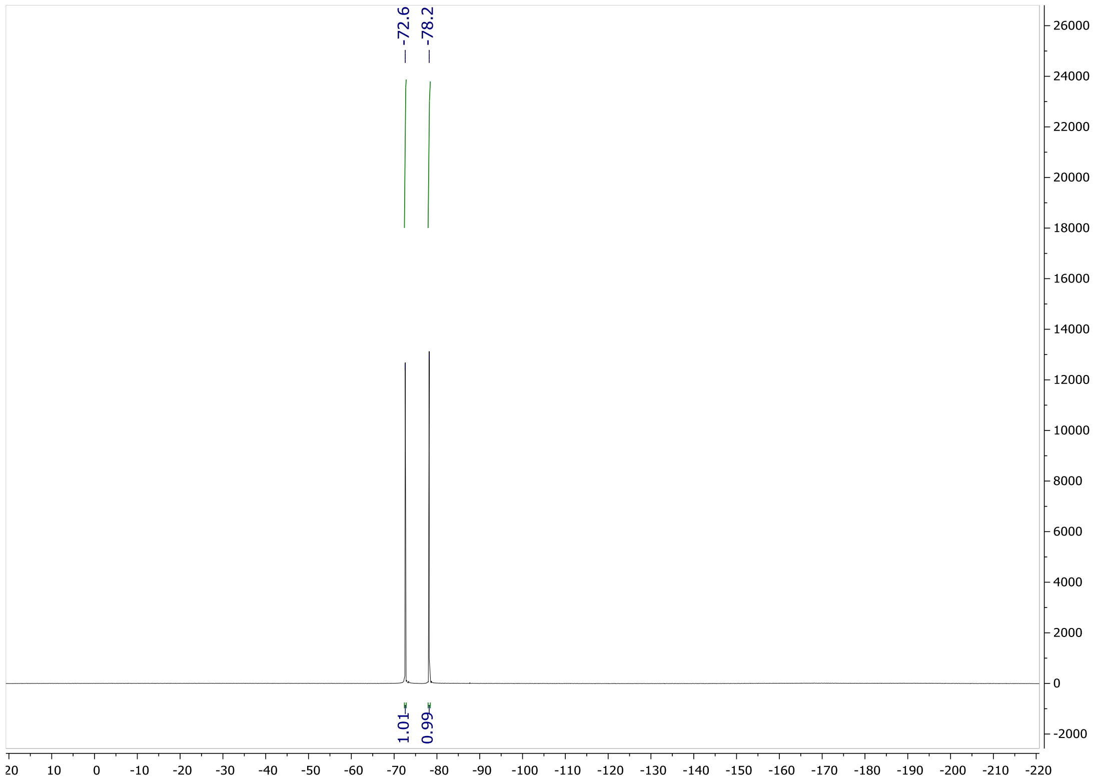

Figure S26. ^{19}F NMR (470 MHz, $CDCl_3$, 16 scans, *T1* = 40 s) of washed **LW-OTf** + TfOH.

13. Fluorescence "turn on" mechanisms and selectivity of LW-OTf and other hemicyanine-xanthene dyes

As illustrated in Scheme 1 and section 4 "Mass spectroscopic analysis" above, it is our understanding that the fluorescent turn-on of probe **LW-OTf** can occur in two ways: 1) Cleavage of the triflyl by superoxide to generate **LW-OH**, and oxidative cleavage of the alkene C=C linker of **LW-OH** to produce **LW-XTD**. The absorption and fluorescence data presented above clearly support the sequential formation of a hemicyanine intermediate followed by a D−π−A xanthene fluorophore. 2) **LW-OTf** can be cleaved to form **LW-XTD-OTf** in presence of $ONOO^-$. This species can then be triflyl-deprotected by superoxide to form **LW-XTD**, which unfortunately could not be confirmed by LC-MS since $ONOO^-$ is prepared in water and KO_2 decomposes in water.[6]

Although the reaction of superoxide with aryl triflate is now a well-established process,[7,8] significant debate remains as to the reaction of peroxynitrite with these types of dyes. In most cases, oxidative cleave of the linker is reported,[9,10] although the exact mechanisms of addition/oxidation has yet to be established. In certain cases, oxidation of the linker is not reported (Scheme S4a and b), although these instances seem predicated on the presence of another highly peroxynitrite-reactive functionality such as the carbonate or selenide.[11,12] One isolated report has suggested that the hemicyanine-xanthene dye undergoes oxidative cleavage, stopping the FRET of **SiRho-HD**, thus leading to FRET "turn-off".[13]

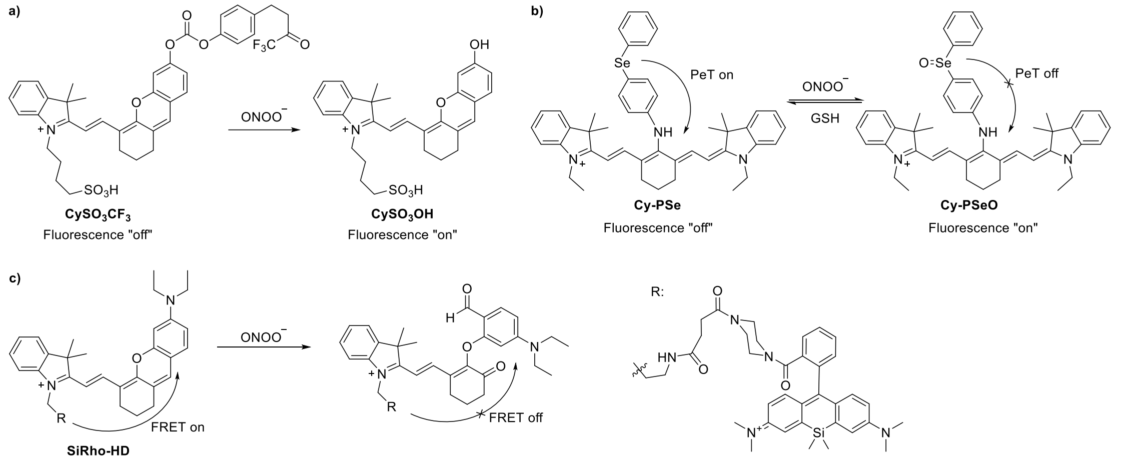

Scheme S4. Alternative fluorescent $ONOO^-$ "turn-on" mechanisms for hemicyanine dyes. (a) Oxidation-deprotection of trifluoromethyl ketone. (b) Organoselenium oxidation for PeT quenching. (c) Xanthene oxidative ring opening for FRET quenching.

14. Statistical analysis

For each experiment, unless otherwise noted, n represents the number of individual biological replicates. For data analysis of NIRF and TPEF images of cells (Fig. 4), we firstly selected regions of interest (ROIs) of 5 images of each group (n=5), then we used Zeiss software to measure fluorescence intensity and output intensity. For data analysis of one-photon fluorescence images of cells (except Fig. 4), we firstly selected ROIs of 5 images of each group (n=5), then we used Leica Application Suite X software to measure fluorescence intensity and output intensity. For data analysis of *in vivo* TPEF images of mice, we firstly selected ROIs of 5 images of each group (n=5, five mice in each group), then we used Zeiss software to measure fluorescence intensity and output intensity. For data analysis of *in vivo* NIRF images of mice, we firstly selected ROIs of 5 images of each group (n=5, five

mice in each group), then we use Living Images software to measure fluorescence intensity and output intensity. All data are expressed as the mean ± S.D. Each experiment was repeated 5 times with identical results.

15. References

1. J. Zhang, C. Li, C. Dutta, M. Fang, S. Zhang, A. Tiwari, T. Werner, F.-T. Luo and H. Liu, *Anal. Chim. Acta*, 2017, **968**, 97-104.
2. E. Alcalde, I. Dinarès, A. Ibáñez and N. Mesquida, *Molecules*, 2012, **17**, 4007-4027.
3. A. Trummal, L. Lipping, I. Kaljurand, I. A. Koppel and I. Leito, *J. Phys. Chem. A*, 2016, **120**, 3663-3669.
4. W. F. Giauque and R. Wiebe, *J. Am. Chem. Soc.*, 1929, **51**, 1441-1449.
5. L. M. Abts, J. T. Langland and M. M. Kreevoy, *J. Am. Chem. Soc.*, 1975, **97**, 3181-3185.
6. F. Tampieri, G. Cabrellon, A. Rossa, A. Barbon, E. Marotta and C. Paradisi, *ACS Sens.*, 2019, **4**, 3080–3083.
7. J. J. Hu, N.-K. Wong, S. Ye, X. Chen, M.-Y. Lu, A. Q. Zhao, Y. Guo, A. C.-H. Ma, A. Y.-H. Leung, J. Shen and D. Yang, *J. Am. Chem. Soc.*, 2015, **137**, 6837-6843.
8. L. Wu, L. Liu, H.-H. Han, X. Tian, M. L. Odyniec, L. Feng, A. C. Sedgwick, X.-P. He, S. D. Bull and T. D. James, *New J. Chem.*, 2019, **43**, 2875-2877.
9. D.-Y. Zhou, Y. Li, W.-L. Jiang, Y. Tian, J. Fei and C.-Y. Li, *Chem. Commun.,* 2018, **54**, 11590–11593.
10. U. N. Guria, A. Gangopadhyay, S. S. Ali, K. Maiti, S. K. Samanta, S. Manna, A. K. Ghosh, M. R. Uddin, S. Mandal, A. K. Mahapatra, *Anal. Methods*, 2019, **11**, 5447-5454.
11. J. Zhang, X. Zhen, J. Zeng and K. Pu, *Anal. Chem.*, 2018, **90**, 9301-9307.
12. F. Yu, P. Li, G. Li, G. Zhao, T. Chu and K. Han, *J. Am. Chem. Soc.*, 2011, **133**, 11030–11033.
13. H.-W. Liu, H. Zhang, X. Lou, L. Teng, J. Yuan, L. Yuan, X.-B. Zhang and W. Tan, *Chem. Commun.*, 2020, **56**, 8103-8106.
